# Ureteric Complications and Urinary Tract Reconstruction Techniques in Renal Transplantation: A Surgical Essay

**DOI:** 10.3390/jcm14124129

**Published:** 2025-06-11

**Authors:** Dorin Novacescu, Hassan Abol-Enein, Silviu Latcu, Flavia Zara, Cosmin-Ciprian Secasan, Vlad Barbos, Victor Pasecinic, Mihael Musta, Ahmad Mohammed Albarakaty, Abdulaziz Bakhsh, Hossam Ismail, Alin Adrian Cumpanas

**Affiliations:** 1 Department II of Microscopic Morphology, Victor Babes University of Medicine and Pharmacy Timisoara, E. Murgu Square, No. 2, 300041 Timisoara, Romania; novacescu.dorin@umft.ro (D.N.); flavia.zara@umft.ro (F.Z.); 2 Urology Department, Urology and Nephrology Center, Mansoura University, Mansoura 35516, Egypt; 3 Department XV, Discipline of Urology, Victor Babes University of Medicine and Pharmacy Timisoara, E. Murgu Square, Nr. 2, 300041 Timisoara, Romania; silviu.latcu@umft.ro (S.L.); cosmin.secasan@umft.ro (C.-C.S.); victor.pasecinic@umft.ro (V.P.); mihael.musta@umft.ro (M.M.); cumpanas.alin@umft.ro (A.A.C.); 4 Doctoral School, Victor Babes University of Medicine and Pharmacy Timisoara, E. Murgu Square, Nr. 2, 300041 Timisoara, Romania; vlad.barbos@umft.ro; 5 Department of Urology, Faculty of Medicine, International Medical Center, Jeddah 23214, Saudi Arabia; albarakatyahmad96@gmail.com; 6 Department of Urology, King Faisal Specialist Hospital and Research Center, Madinah 42522, Saudi Arabia; abksh@taibahu.edu.sa; 7 Department of General and Specialized Surgery, College of Medicine, Taibah University, Madinah 42353, Saudi Arabia; 8 Department of Urology, Lausitz Seenland Teaching Hospital, University of Dresden, Maria-Grollmuß-Straße, No. 10, 02977 Hoyerswerda, Germany; drismailhossam@gmail.com

**Keywords:** kidney transplantation, surgical challenges in urinary reconstruction, ureteric complications, strictures and urinary leaks, post-transplant care, long-term outcomes, ureteroneocystostomy, native upper tract anastomosis, ureteroenterostomy, pyeloureterostomy and ureteroureterostomy

## Abstract

**Background/Objectives**: Renal transplantation (RT) remains the gold standard for end-stage renal disease, offering superior outcomes versus dialysis. Despite advances, ureteric complications (leaks/strictures) persist, primarily from ischemic injury, posing substantial graft risks. We review etiology, incidence, and management strategies for post-RT ureteric complications, focusing on surgical reconstruction techniques. **Methods**: Literature assessment examined ischemic-related ureteric complications. Primary outcomes: incidence, success, complication rates, operative times, and long-term patency. Secondary outcomes: graft/patient survival and reoperation rates. Techniques evaluated included extravesical Lich–Gregoir (L-G) and transvesical Leadbetter–Politano (L-P) ureteroneocystostomy (UNC), Boari flap with psoas hitch, pyelo/ureteroureterostomy, pyelovesicostomy, and ureteroenterostomy. Surgical indications, procedural details, advantages, disadvantages, and quantitative outcomes were systematically analyzed. **Results**: Ureteric complication incidence ranged from 1 to 15%, with ischemic injury as the primary cause. L-G UNC demonstrated lower complication rates than L-P (6.15% vs. 8.33%) with reduced operative times. Pyelo/ureteroureterostomy achieved excellent salvage outcomes (>90% success, 3.9% reintervention rate). Boari flap provides a suitable option for extensive ureteric defects, consistently preserving graft function without stricture recurrences. Pyelovesicostomy showed 80% long-term success in complex cases. Ureteroenterostomy achieved comparable 5-year graft survival (63%) to standard drainage, despite higher infection rates (65%). Pyelovesicostomy and ureteroenterostomy remain important solutions for specific challenging scenarios. **Conclusions**: Urinary reconstruction technique selection should be individualized based on anatomical considerations, pathology, and surgical expertise. Comprehensive understanding of reconstruction techniques enables effective management of ureteric complications, preserving graft function and improving outcomes.

## 1. Introduction

Renal transplantation (RT) remains the preferred treatment for end-stage renal disease, offering improved survival and quality of life compared to dialysis [1]. However, urologic complications represent significant challenges for recipients, with substantial implications for graft survival and patient outcomes. While impossible to eliminate entirely, these complications can be minimized through appropriate prevention strategies, timely diagnosis, and effective management protocols.

The reported incidence of urologic complications post-RT ranges widely (1–15% [2,3]) and has remained relatively stable despite surgical technology advances. This variability derives from differences in follow-up duration and definition breadth. Some studies include hematuria, urinary tract infection (UTI), and urinary retention, while others focus specifically on ureteric strictures or leaks. Ureteric complications at the vesicoureteric anastomosis or along the transplanted ureter adversely affect graft function and remain significant sources of post-RT morbidity [3].

Ureteric leaks and strictures occur in ~2–5% of RT recipients [1,2], with fistulas being the most frequent first-month complication [1]. Early fistulization (≤day 4) typically results from technical issues, while later leaks (≥day 10) are associated with ischemic necrosis. The underlying cause is often ischemia of the distal donor ureter, uniquely vulnerable due to sole blood supply from inferior renal artery branches [2]. Ischemic injury leads to ureteric necrosis, fistula formation, or fibrotic scarring and stricture development later on, threatening graft function and requiring prompt surgical management [2,3].

In fact, multiple factors contribute to post-RT ureteric complications. Technical factors are paramount—meticulous surgical technique preserves ureteric blood supply, while excessive periureteric tissue dissection causes devascularization and ischemia. Herein, patient and donor factors also play a role in the incidence of urinary complications. Risk factors include delayed graft function (DGF), donor age > 65 years, repeat RT, male recipients, obesity, multiple renal arteries, and poor bladder condition [3]. Protective factors include living donor transplants (shorter ischemia times, careful harvesting) and routine ureteric stenting [3,4]. Despite declining immunologic graft loss, ureteric problems remain important causes of post-RT morbidity [3,4].

Urinary tract reconstruction in RT typically involves implantation of the graft ureter into the recipient bladder, i.e., ureteroneocystostomy (UNC). Several techniques exist for this anastomosis, each with particular advantages and potential drawbacks. In addition, more complex, acute or delayed complications may require alternative reconstructive strategies beyond the standard UNC. This narrative review will discuss the major ureteric complications following RT—specifically ureteric leaks and strictures as results of ischemic injury to the ureter—and examine the surgical techniques for urinary tract reconstruction employed to treat and/or prevent these issues—from the standard extravesical Lich–Gregoir (L-G) and trans/intravesical Leadbetter–Politano (L-P) UNC techniques to more particular approaches like the Boari flap (with psoas hitch), pyeloureterostomy, pyelocystostomy and ureteroenterostomy. A comparative analysis of these techniques, highlighting their advantages, disadvantages, and outcomes, is provided. Relevant postoperative management strategies and potential complications associated with each approach are reviewed, with an emphasis on optimizing graft outcomes and patient safety. The goal is to inform surgical decision-making and highlight best practices for managing ureteric issues in RT patients.

## 2. Ureteric Complications After Renal Transplantation

Overall, urologic complications occur in a minority of kidney transplants, but their impact can be significant. One large single-center study reported an overall incidence of 7.8%, with ureteric stenosis (3.1%) and urine leakage (2.4%) being the most common issues [4]. Nearly 40% of these complications occurred within the first month post-RT [4], underlining that the early postoperative period is high-risk, especially for leaks. We discuss each major complication below, along with its causes and management considerations.

### 2.1. Ureteric Ischemia and Its Pathogenic Role

Ischemia of the transplanted graft ureter represents the fundamental factor underlying most ureteric complications. Unlike a native ureter, which has a rich, lengthy adventitial blood supply from multiple segmental arteries along its course (i.e., the upper proximal part directly from the inferior renal artery; the middle lumbar part from abdominal aortic branches and the common iliac and gonadal arteries; and the inferior distal part from internal iliac artery branches), the graft ureter relies almost exclusively on blood flow from the inferior renal artery branches near the renal hilum. These small arterial branches travel through the periureteric fibro-fatty tissue, particularly in an area known as the “golden triangle”, adjacent to the lower pole of the kidney. If this delicate blood supply is compromised—for example, by excessive stripping of periureteric tissue during organ retrieval or bench preparation—the distal ureter may become ischemic. Indeed, even in the absence of obvious technical errors at the time of implantation, ischemia and necrosis of the distal ureter are considered the primary causes of early ureteric complications such as urinary fistulas [5].

The importance of preserving the ureter’s surrounding tissue cannot be overstated: damage to the lower polar artery or its branches in the graft’s periureteric tissue greatly increases the risk of ureteric necrosis [5]. Even with meticulous technique, the distal few centimeters of the donor ureter often have tenuous blood flow. If the ureter is left too long or if there is tension/kinking in the UNC site, the distal segment may become necrotic. Ureteric ischemia typically manifests within the first 1–3 weeks postop as either a urinary fistula, i.e., ureteric leakage, or later on, as a ureteric stricture. In fact, most early ureteric complications can be viewed as a spectrum of ischemic injury severity—complete necrosis yields a fistula (leak), whereas partial ischemia leads to scarring and stenosis [1,2].

Inherently, preventing ischemia is a key principle of the UNC. Surgical measures to mitigate ischemic risk include using the shortest necessary length of ureter and preserving its adventitial tissue [6]. Surgeons typically avoid skeletonizing the donor ureter; a “cuff” of periureteric tissue, in continuation of the renal pelvis, and even a patch of donor bladder in some techniques [7], are kept intact to safeguard blood flow. Gentle handling and avoiding excessive diathermy near the ureter are also essential. Moreover, the distal end of the ureter is often trimmed to a well-vascularized segment and spatulated to ensure a wide, tension-free, mucosal anastomosis. This preparation helps ensure good perfusion at the distal ureteric tip, minimizing the chance of ischemic strictures or breakdown of the anastomosis [5].

Furthermore, some centers routinely place a prophylactic ureteric stent, theorizing that it supports the anastomosis and mitigates urine leakage if minor ischemic sloughing occurs; stents may also allow a partially compromised anastomosis to remain patent while healing. However, stents come with tradeoffs (infection, encrustation), and practices vary. Notably, one high-volume transplant center reported using an extravesical L-G technique without any stenting as their routine protocol, reserving stents only for more complicated cases [8]. In their experience, strict attention to ureteric blood supply and delicate surgical technique yielded acceptable leak/stricture rates even without stents [8]. Thus, preventing ureteric ischemia hinges on surgical technique and intraoperative judgment.

In summary, distal ureteric ischemia represents the pathogenic lesion that links many ureteric leaks and strictures post-RT; prevention hinges on meticulous surgical technique that maintains blood supply to the ureter, both during harvesting and implantation. When ischemia does occur, early recognition and intervention are critical, i.e., conservative measures and/or surgical revision of the urinary anastomosis. If the distal ureter is necrotic, a creative solution is required to bridge the gap between the kidney graft and the recipient bladder or native ureter. The following sections review the repertoire of urinary tract reconstruction techniques that the RT surgeon may employ, both during the initial graft implantation and as salvage procedures for ischemic complications post-RT.

### 2.2. Epidemiology and Risk Stratification

Contemporary multi-center analyses demonstrate variable but consistent patterns in ureteric complication rates across transplant programs. Large population-level studies, including an analysis of >9000 patients, report overall urologic complication rates of 11.3% [9], with significant variation based on institutional protocols and patient populations [3]. High-volume centers employing standardized surgical protocols and routine stenting typically achieve lower complication rates in the 4–5% range, compared to 7–15% in centers with variable practices [4,6]. Thus, institutional experience significantly influences outcomes, with specialized transplant centers reporting lower complication rates through systematic quality improvement initiatives. Inherently, centers implementing standardized procurement protocols, routine ureteric stenting, and structured surgical training programs achieve superior outcomes compared to those with variable practices. This institutional effect underscores the importance of specialized expertise and systematic approaches in minimizing ureteric complications.

Multi-institutional data consistently identify key risk factors for ureteric necrosis in post-RT, associated with higher complication rates across diverse patient populations. Understanding patient-specific risk factors enables proactive surgical planning and technique selection. High-risk profiles requiring enhanced surgical vigilance and potentially alternative techniques include specific donor, recipient, and technical factors that significantly influence complication rates.

Donor-related risk factors center on vascular compromise and tissue quality. Advanced donor age (>65 years) is associated with increased atherosclerotic disease affecting the critical periureteric vasculature, i.e., compromised micro-perfusion [10]. Prolonged cold ischemia time (>20 h) compounds ischemic risk, particularly affecting the vulnerable distal ureter—which is also concomitantly undergoing the mechanical stress of surgical reimplantation into the recipient’s bladder. Conversely, donation after cardiac death grafts carry higher complication rates due to warm ischemia exposure. Moreover, grafts with multiple renal arteries, requiring complex vascular reconstruction, may compromise collateral ureteric blood supply during surgical manipulation [3,11,12]. Finally, severe DGF, acute rejection (AR) or pyelonephritis can also further exacerbate injury to an already marginal ureteric blood supply [1,3]. Corroborating this claim, a single-center study by Karam et al. (2004) reported that ureteric necrosis occurred in only 3.2% of kidney transplants (out of 1629 RT cases analyzed), yet it was associated with DGF and AR episodes while also significantly impacting overall graft survival when not promptly addressed [10].

Regarding recipient factors influencing surgical outcomes, diabetes mellitus stands out, impairing wound healing and increasing infectious risks, thereby potentially complicating anastomotic healing. Furthermore, obesity (body mass index > 30) creates additional technical challenges due to the increased depth of surgical fields and, implicitly, the subsequent increase in tissue tension during implantation. Conversely, previous pelvic surgery, radiation therapy, or peritoneal dialysis-related adhesions further complicate surgical access and increase the likelihood of requiring alternative reconstruction techniques. Neurogenic bladder disorders or bladder augmentation necessitate specialized approaches and careful preoperative urodynamic assessment [5].

Technical factors remain under direct surgeon control and significantly influence outcomes. Excessive ureteric dissection during procurement devascularizes the critical periureteric tissue. Inadequate ureteric length or tension on the anastomosis increases ischemic risk. Suboptimal surgical technique, including inadequate spatulation or improper suture placement, directly correlates with higher complication rates. Recognition of these factors allows proactive technique modification and risk mitigation strategies.

This risk stratification framework enables individualized surgical planning, with high-risk cases potentially benefiting from alternative primary techniques or enhanced perioperative monitoring protocols.

### 2.3. Ureteric Leaks (Urinary Fistulas)

Urine leaks represent serious early complications after RT. A ureteric leak refers to extravasation of urine due to a failure of the ureteric anastomosis or a defect in the ureter. Ureteric leaks typically present within the first days or weeks postoperatively, often while the patient is still hospitalized or soon after discharge. Patients may have fluid accumulation in the transplant bed or draining from the operative site through the surgical drain, with elevated creatinine in the drain fluid, diminished urine output, or signs of sepsis if infection ensues [11].

Clinically, a significant leak may present with abdominal pain, swelling, ileus, or a persistently high drain output of clear fluid. Because this fluid could be lymph or urine, a key diagnostic step is to analyze the drain fluid for creatinine concentration. Fluid that has a high creatinine level confirms a urine leak from the urinary system. Most leaks tend to occur at the distal end of the graft ureter, predominantly at the UNC site [3,11].

Early leaks (within the first 4–7 days) are often due to technical issues at the anastomosis (e.g., suture failure), whereas “late” leaks (around 7–10 days postop) are more commonly associated with ischemic necrosis of the distal ureter [2]. The incidence of post-RT urinary leakage is reported in the range of ~3–4% [12], although large centers with routine stenting may achieve lower rates. Prompt diagnosis is critical, often confirmed by ultrasound or computer tomography (CT) showing perinephric fluid collection (urinoma) and/or a retrograde renogram/cystogram, usually demonstrating extravasation of contrast at the level of the urinary anastomosis (UNC) [11].

Ischemic leaks are the most common type, typically caused by necrosis of the distal donor ureter due to insufficient blood supply, as the donor ureter receives blood only from the renal artery, and any surgical devascularization or tension can compromise distal perfusion [1]. Risk factors for ureteric necrosis include lengthy donor ureters, damage to the periureteric tissue during procurement, or prolonged ischemia times. When the distal ureter undergoes necrosis, a urinary fistula can form at the bladder anastomosis or along the ureteric stump. In one series, ureteric fistulas were noted in about 2–4.7% of transplants, almost always tied to distal ureteric ischemia [1].

Once a leak is confirmed, management depends on its severity. Minor contained leaks or urinomas might be managed conservatively, with minimally invasive measures to obtain maximal decompression, i.e., prolonged bladder drainage (keeping the Foley catheter in place) and ureteric stenting to divert urine away from the leak, giving it a chance to heal. In fact, if a difficult or fragile anastomosis was created, many surgeons will intentionally leave the Foley catheter for an extended period and only remove it after performing a cystogram to ensure no leak is present [13]. Thus, a fully diverting ureteric stent and low bladder voiding pressures can allow minor anastomotic leaks to seal. However, large leaks with extensive extravasation, large proximal fistulas, or leaks that do not respond to maximal decompression will usually require surgical exploration and urinary reconstruction to re-establish a secure urinary outflow [1].

Initial options include redoing the UNC, i.e., reimplanting the donor ureter into the bladder at a new site (if sufficient length remains), or performing a ureteroureterostomy/pyeloureterostomy into a native recipient ureter if the transplanted ureter is (mostly) unsalvageable (discussed in detail below). In cases of extensive distal ureteric necrosis, simply redoing the UNC may not be feasible, and more complex reconstructions like a Boari flap or pyelovesicostomy might be indicated. Endoscopic approaches (percutaneous nephrostomy with antegrade stenting) have a role in temporizing the situation or in high-risk patients; however, purely endourologic management of significant transplant ureteric leaks has a limited long-term success rate (~58% in one series, with many failures due to recurrent UTIs or stricture) [10]. Therefore, surgical reconstruction is often the definitive solution for durable correction of ureteric leaks [1].

Prompt recognition and surgical management of ureteric leaks are critical, as ongoing urinary extravasation can lead to infection, abscess, uro-sepsis, or jeopardizing the graft, i.e., dysfunction due to urinoma compression. Encouragingly, with routine use of ureteric stents and improved surgical technique, the incidence of ureteric leaks has been generally reported as being low (around 2–3% or less) in contemporary series [4].

### 2.4. Ureteric Strictures

Ureteric strictures typically occur later in the postoperative course (weeks to years post-RT). A stricture constitutes a narrowing, usually either at the UNC site or within the distal graft ureter, that impedes urine drainage and may thus cause graft hydronephrosis. The incidence of ureteric stenosis in RT patients ranges from ~2% up to 10–13% in various series [14]. Improved surgical techniques and routine stenting have brought incidence rates down, towards the lower end of this range, in modern practice (roughly 2–5%) [6,14]. Strictures share the same fundamental etiology as leaks—ischemia is the primary culprit. If the ureteric blood supply is marginal, the healing process can result in fibrotic narrowing rather than a full-thickness disruption. Other contributors may include technical issues (e.g., a misaligned suture line causing scarring) or prolonged external compression (from peri-graft hematoma or lymphocele). Notably, lymphoceles can form adjacent to the ureter, albeit rarely, and cause extrinsic ureteric compression, presenting similarly to a stricture [6]. Less commonly, recurrent AR episodes producing ureteritis or ureteric cytomegalovirus infections [15,16] have been implicated in late stricture formation, but ischemic fibrosis at the level of the anastomosis remains the predominant mechanism.

BK polyomavirus represents a distinct and particularly challenging etiology of post-RT ureteric strictures that demands special consideration due to its unique clinical characteristics and poor response to conventional management. Similarly to cytomegalovirus-associated ureteric complications [15,16], BK virus-associated strictures typically present months to years following RT, with insidious graft dysfunction often developing in the setting of BK viremia or nephropathy. These strictures characteristically involve long segments of the mid-ureter, extending well beyond the typical distal location of ischemic complications, and demonstrate extensive fibrotic involvement that distinguishes them from other etiologies [17].

The pathogenesis involves viral-induced inflammation and subsequent fibrotic healing that creates dense, lengthy strictures, notoriously resistant to endoscopic intervention. Unlike ischemic strictures that may respond to balloon dilation or endoureterotomy [18], BK-associated lesions demonstrate high recurrence rates and often progress, despite initial technical success of endoscopic procedures [19]. The inflammatory nature of these strictures frequently involves extensive periureteric fibrosis that complicates surgical reconstruction and may require extensive ureteric excision with alternative drainage methods, such as pyeloureterostomy or UNC with psoas hitch [14,20,21]. Recognition of BK virus as a causative factor influences both treatment planning and surveillance strategies, with regular monitoring enabling early detection and potential intervention before irreversible ureteric damage occurs [17].

In fact, recipients with graft ureteric strictures often present with asymptomatic rising serum creatinine or hydronephrosis, noted during routine ultrasound surveillance. Some may experience flank pain or febrile UTIs if the hydronephrosis is more severe. If a stricture is suspected, the initial management is to relieve the obstruction, as immediate decompression of the kidney graft is important to prevent loss of function. This can be achieved either by placing a nephrostomy tube percutaneously or via retrograde placement of a ureteric stent across the stricture, if possible. This not only protects the kidney but also allows assessment of stricture length and location fluoroscopically, i.e., retrograde pyelogram or anterograde nephrostogram [3,22].

Very short, simple strictures at the bladder anastomosis can sometimes be treated with endoscopic balloon dilatation or endoureterotomy, with stent placement for a few weeks. However, long-term, purely endoscopic management of graft ureteric strictures has a high failure rate—balloon dilations or repeated stenting often provide only temporary benefit [18]. Therefore, many of these post-RT strictures will ultimately require a surgical reconstruction for definitive repair [1].

Surgical options mirror those for ureteric leaks: a simple revised UNC (reimplanting the graft ureter into the native bladder at a new site, if enough healthy ureter remains viable after excising the scarred segment) or conversion to an alternative drainage method, as detailed in later sections. In more complex or lengthy strictures, especially those not amenable to a tension-free re-UNC, an open surgical reconstruction is the best option for lasting success [19]. One effective solution is a Boari flap UNC, where a flap of bladder wall is tubularized and hitched up to bridge the gap to the remaining ureter or renal pelvis (discussed in detail later). This approach provides a well-vascularized bypass for the obstructed ureter and has excellent long-term outcomes in the RT setting [20]. In a series of 10 RT cases with ureteric strictures undergoing Boari flap reconstruction, allograft function was preserved in all cases, with over one-year follow-up documenting no stricture recurrence [21].

Other reports similarly describe high success rates with open surgical repair of post-RT ureteric strictures, using other techniques as well [17]. Notably, a systematic review of stricture surgical management strategies found that performing a pyeloureterostomy (attachment of the graft pelvis to the native ureter) was among the most successful open surgical techniques for distal strictures, with high patency rates [2]. Success rates for surgical correction of post-RT ureteric strictures are generally excellent, often >90% in experienced centers [14]. Once the obstruction is resolved, the transplanted kidney usually recovers function, though prolonged unrecognized obstruction can inflict irreversible damage. Thus, while ureteric strictures remain a troublesome complication, prompt surgical management will fortunately lead to excellent outcomes [23,24].

In summary, ureteric complications (leaks or strictures) in RT are usually consequences of ureteric ischemia or technical missteps. Vigilant postoperative monitoring is needed to detect them early on. The principles of management are to secure urinary drainage (via stents or nephrostomy) and then address the underlying issue definitively, often with open surgical revision or reconstruction. Preventing these complications begins in the operating room—which brings us to the surgical techniques used for ureteric implantation and how they impact ureteric complication rates.

### 2.5. Pre-Reconstruction Urological Assessment and Workup

Comprehensive urological preoperative evaluation identifies recipients at high risk for post-RT urinary complications, enabling proactive management strategies that significantly improve outcomes. Patients with neurogenic bladder disorders, congenital urological anomalies, or previous urinary reconstruction require specialized assessment and often anticipatory intervention. The prevalence of complex urological conditions among ESRD patients, particularly those with diabetes mellitus or spina bifida, necessitates systematic evaluation protocols [3,5].

Urodynamic studies should be considered in recipients with neurogenic bladder disorders of any etiology, congenital anomalies such as spina bifida or posterior urethral valves, previous bladder surgery or augmentation, recurrent UTIs suggesting poor bladder dynamics, suspected detrusor dysfunction or bladder outlet obstruction, as well as when complex reconstruction planning requires detailed knowledge of bladder behavior [24]. These studies provide essential information about bladder capacity, compliance, detrusor pressures, and voiding dynamics (residual volumes and leak point pressures) that directly influence reconstruction technique selection. Low bladder capacity (<200 mL), poor compliance (<10 mL/cm H_2_O) or high detrusor pressures during filling (>40 cm H_2_O), indicate high-risk scenarios requiring aggressive optimization before reconstruction to prevent pressure-mediated graft damage [3,5,9].

Bladder optimization strategies must address both functional and infectious considerations. Medical management includes anticholinergic therapy for detrusor hyperactivity, establishment of clean intermittent catheterization protocols for incomplete voiding, and botulinum toxin injection for medically refractory detrusor hyperactivity. When conservative measures fail to achieve safe bladder dynamics, surgical intervention such as bladder augmentation or creation of continent diversions may be necessary before proceeding with reconstruction. Concurrent management of UTIs through culture-directed antimicrobial therapy and optimization of immunosuppressive regimens when possible helps minimize perioperative infectious complications [5,12].

The decision between anticipatory and reactive urological intervention depends on individual patient factors and bladder dynamics. Primary anticipatory reconstruction benefits patients with small-capacity bladders, high-pressure voiding systems, significant vesicoureteric reflux (VUR), or documented recurrent pyelonephritis [9]. Patients with spina bifida often require pre-transplant bladder augmentation to create a safe, low-pressure reservoir capable of protecting the future graft. Similarly, recipients with neurogenic bladder dysfunction may benefit from the establishment of catheterizable channels or other specialized drainage procedures before RT. This proactive approach contrasts with reactive management, where urological intervention occurs only after complications develop, often resulting in more complex reconstructions and increased morbidity [3,5].

Systematic evaluation before undertaking urinary reconstruction determines the optimal surgical approach and predicts potential outcomes in post-RT patients with urologic complications. The diagnostic workup must comprehensively assess graft function, define anatomical relationships, evaluate recipient bladder dynamics, and identify factors that might influence reconstruction success [17,22]. Imaging studies form the cornerstone of assessment. Initial ultrasound evaluation (with Doppler signal analysis) assesses hydronephrosis, identifies fluid collections, and evaluates graft perfusion patterns [11]. Urographic computer tomography provides detailed anatomical definition, including precise stricture localization, assessment of surrounding inflammation, and identification of anatomical variants that might influence surgical planning [17,22]. Nuclear medicine renography quantifies differential graft function and drainage patterns, providing objective data about functional impact and potential for recovery following reconstruction [11,22]. Retrograde pyelography remains the gold standard for defining ureteric anatomy, characterizing stricture length and complexity, and planning the extent of surgical intervention required [17,22].

Lastly, intraoperative identification of the native ureter during revision surgery can prove challenging in the presence of inflammation, fibrosis, and anatomical distortion. Cystoscopic placement of a retrograde ureteric stent, under fluoroscopic guidance, 24 to 48 h before planned reconstruction significantly facilitates surgical dissection and reduces the risk of inadvertent ureteric injury [4,22]. If retrograde access proves impossible due to obstruction or anatomical factors, percutaneous antegrade stenting provides equivalent benefits for surgical planning and intraoperative identification [11,22]. This preparatory step proves particularly valuable in cases requiring extensive dissection or when anatomical landmarks have been obscured by previous surgery or inflammation [19].

## 3. Recipient Bladder Anastomoses

Following vascular anastomoses and graft reperfusion, urinary reconstruction becomes critical. Creating a reliable urinary tract anastomosis is a key component of RT surgery. The standard approach is to implant the donor ureter into the recipient’s bladder, i.e., UNC. Several techniques for UNC exist, with the choice depending on the length and condition of the donor ureter and the specifics of the recipient’s anatomy. The two principal contemporary UNC techniques are the extravesical L-G and the transvesical L-P, employed in the vast majority of kidney transplants for urinary reconstruction. Conversely, the extravesical L-G technique (or one of its variations) is generally preferred and more prevalently employed. This approach advantageously avoids additional cystotomy, requires shorter donor ureters, and offers simplicity and versatility while reducing operative times and bladder-related complications, including hematuria, urinary retention, and fistulas [23].

Both UNC methods preserve the native ureter for future management of potential complications [24]. When standard approaches prove unfeasible—due to extremely short or damaged donor ureters—alternative reconstructive methods become necessary, including Boari flap UNC (sometimes with psoas hitch) or ureteroureterostomy (graft-to-native ureter anastomosis). In complex scenarios involving prior bladder augmentation or urinary conduits, careful planning is essential, often employing protective indwelling stents to ensure optimal postoperative drainage and graft function [24,25]. Below, we describe each technique in turn and provide a comparative discussion of their individual merits and outcomes.

### 3.1. Extravesical Lich–Gregoir Ureteroneocystostomy

The extravesical L-G UNC is currently the most commonly performed urinary anastomosis in RT. In this technique, the bladder cavity is not opened; instead, the donor ureter is anastomosed to the bladder detrusor from the outside (extravesically), creating a submucosal tunnel to provide a valve mechanism [26,27,28]. Herein, it is worth noting that the tunnel length in extravesical repair is shorter than with an intravesical approach [24]. Even so, the entire anastomosis is performed without making a formal cystotomy (no full-thickness bladder incision). Placement of a JJ stent (5Ch, 12 cm) across the anastomosis is at the surgeon’s discretion but is recommended to decrease postoperative complications, although concerns about reflux-related infections persist [29,30,31].

#### 3.1.1. Surgical Technique

The transplant ureter is generally routed posteriorly to the spermatic cord or round ligament to avoid kinking and potential obstruction. In some cases, division of the spermatic cord may facilitate ureteric placement without significant long-term complications [29]. The donor ureter is tailored to the appropriate length, ensuring tension-free anastomosis, and spatulated to provide a wide opening and maintain distal perfusion, mitigating ischemic complications [29,32]. In order to facilitate handling and the dissection of its individual parietal layers, the bladder is distended with approximately 150–200 mL of saline antibiotic solution via a Foley catheter inserted preoperatively (and fitted with a Y-connector for additional control and versatility) [33]. Alternatively, methylene blue can be added to this irrigation, as it may prove useful in more clearly differentiating between the bladder wall and the peritoneum, particularly in obese recipients with pelvic scarring (peritoneal dialysis) or reduced bladder capacity [32,33].

**Figure 1:**
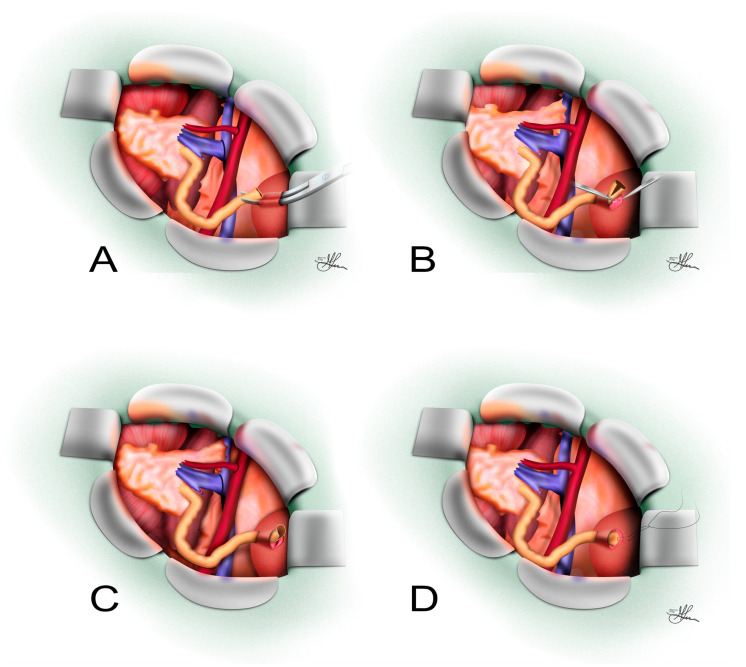
Extravesical Lich–Gregoir ureteroneocystostomy surgical technique: (**A**) two small parallel sero-muscular incisions are made in the donor bladder wall, perpendicular to the planned ureteric coarse, and a submucosal tunnel is dissected, to pass the donor ureter through; (**B**) the bladder mucosa, bulging at the distal end of the submucosal tunnel, is incised and 5–10 mm opening is created to allow for the anastomosis; (**C**) the spatulated ureteric stump is brought to the bladder mucosa and the toe end of the ureter is secured to the full thickness of the bladder wall; (**D**) a mucosa-to-mucosa anastomosis is performed, using a fine slow-absorbable monofilament suture (e.g., 5-0 polydioxanone), usually in a running manner, between the full thickness of the ureter and the dissected bladder mucosa, at the level of the opening created. N.B.: original medical illustrations created by Marius Filip using professional graphic design software.

In the L-G technique (see Figure 1), a small incision is made in the sero-muscular layer of the bladder’s anterior or lateral wall (usually on the superior lateral aspect) for the ureteric implant. There are variations in how this incision is made. The traditional approach is to make two small (~1.5 cm) parallel incisions in the detrusor, perpendicular to the ureter’s planned direction [34], causing the bladder mucosa to bulge at the incision sites and allowing for the dissection of a short trough to pass the ureter through (see Figure 1A) [33]. Another common approach is a single 2.5–3 cm incision in the bladder detrusor muscle (going in the direction of the planned ureteric course) [35]. The detrusor muscle fibers are gently separated to fashion a submucosal tunnel about 1–2 cm in length.

In either case, the idea is to create a tunnel in the bladder wall. At the distal end of this tunnel, the bladder mucosa is opened (~10 mm diameter opening) to allow ureteric entry into the bladder lumen (see Figure 1B). The donor ureter, which has been spatulated (split longitudinally at its end) to create a wide opening for the anastomosis, is then brought to the bladder mucosa (see Figure 1C). The toe end of the ureter is secured to the full thickness of the bladder wall (see Figure 1C) [36]. A mucosa-to-mucosa anastomosis is performed between the ureter and the bladder mucosal opening. Typically, a fine, slow-absorbable monofilament suture (e.g., 5-0 polydioxanone/PDS) is used, usually in a running manner, to sew the full thickness of the ureter to the bladder mucosa (see Figure 1D) [32]. Only the mucosa (and a small rim of ureteric wall) is incorporated in the anastomosis, avoiding deep bites that could occlude the lumen or damage the blood supply [32].

Once the ureter is anastomosed, if the single incision technique was used, the detrusor muscle flaps created are reapproximated over the ureter using interrupted absorbable sutures (2-0 or 3-0 polyglactin/Vicryl), creating a protective submucosal tunnel. Care is taken to avoid over-tightening this tunnel to prevent ureteric obstruction [24,32]. Similarly, the distal incision is closed over the anastomosis in the traditional technique. In many cases, especially in adult transplant recipients, creating a long anti-reflux tunnel is not necessarily a main priority, seeing as the extravesical anastomosis essentially results in a shortened intramural course yet is still the more prevalent technique used. Nonetheless, when performed properly, the L-G implant does confer a degree of anti-reflux because the ureter is still surrounded by the detrusor backing and the angle of entry is oblique [37].

#### 3.1.2. Indications and Outcomes

The L-G method is favored for its simplicity and speed. It avoids a bladder incision and hence reduces operative time and blood loss from the bladder [37]. It also requires a shorter length of donor ureter, since the ureter does not need to traverse deep into the bladder lumen [6]. This is advantageous when the donor ureter is marginal in length or blood supply. Additionally, by not opening the bladder, the risk of bladder-related complications such as prolonged hematuria or bladder leak is minimized. Many surgeons find the extravesical approach technically straightforward, and it can be performed even in the presence of a distended bladder (where transvesical access might be more challenging) [37].

Importantly, contemporary studies have shown that the L-G extravesical technique achieves low rates of ureteric complications, comparable to (or even lower than) intravesical techniques [6,23,37]. In the largest Romanian single-center comparative series of 524 transplants, the extravesical L-G technique had a slightly lower overall urologic complication rate (6.15%) than the L-P transvesical method (8.33%), although the difference was not statistically significant [6]. Specifically, ureteric stenosis occurred in 2.3% with L-G vs. 3.4% with L-P, and leak rates were similar [6]. Graft and patient survival did not differ between the two techniques [6]. These findings support the safety of the extravesical approach. Surgeons also note that extravesical reimplantation spares the patient a cystotomy, which can be beneficial in terms of postoperative pain and allows earlier regain of bladder functionality [37].

The main concern historically raised about the L-G technique is a potentially higher incidence of VUR, since the submucosal tunnel is shorter. Reflux in a transplanted kidney could, in theory, predispose to pyelonephritis. However, many centers report that clinically significant reflux is not a frequent problem with a well-executed extravesical reimplant, especially when a stent is used to support the anastomosis during healing. Moreover, reflux into a transplanted kidney might be less problematic than in native systems, as transplant ureters are often narrowed and the allograft kidney is routinely monitored, i.e., any pyelonephritis is promptly treated. Another limitation is that the extravesical technique creates a fixed position for the ureteric orifice; some surgeons feel this approach gives slightly less freedom to adjust the ureter’s course, which in rare cases could result in a kink if the transplant kidney shifts [23,37]. Overall, however, the L-G approach is considered to have few disadvantages and is widely regarded as the technique of choice for most kidney transplant UNCs.

### 3.2. Transvesical Leadbetter–Politano Ureteroneocystostomy

**Figure 2:**
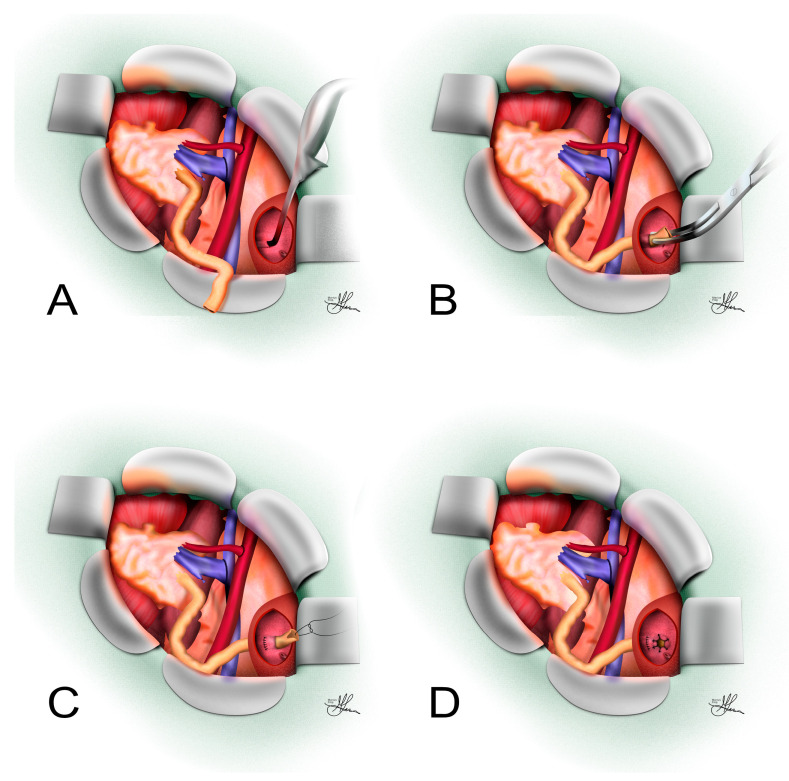
Transvesical Leadbetter–Politano ureteroneocystostomy surgical technique: (**A**) Through an anterolateral cystotomy, a separate oblique full-thickness posterior parietal stab incision is performed, superolateral to the ipsilateral native ureteric orifice, and then additionally dilated using a tunneling tool; (**B**) The ureter is then guided through the tunnel, into the bladder; (**C**) An additional mucosal incision can be performed (2 cm apart), to allow for additional tunnelization of the ureteric stump under the mucosa, which will then be prepared (transected and spatulated) and used for placing the inferior sutures of the anastomosis, i.e., incorporating bladder muscle to anchor the ureter securely within the submucosal tunnel; (**D**) The spatulated ureter is anastomosed to the bladder mucosa around the newly developed ureteric orifice, using fine absorbable sutures. N.B.: original medical illustrations created by Marius Filip using professional graphic design software.

The classic intravesical UNC techniques were originally described as an approach for ureteric reimplantation to treat VUR in children, i.e., Cohen cross-trigonal reimplantation [38]. In transplant surgery, the transvesical L-P technique involves creating a full-thickness parietal opening into the bladder cavity (cystotomy) and implanting the donor ureter through the bladder wall, within a transparietal submucosal tunnel, created from the mucosa outward (see Figure 2).

#### 3.2.1. Surgical Technique

The standard protocol for transvesical L-P UNC closely resembles the original technique, described by Merrill and colleagues, during the first successful kidney transplant, performed between identical twins [39]. Typically, the bladder is opened anteriorly or laterally, and the donor ureter is passed endovesically, using a separate oblique stab incision through the posterior bladder wall (often superolaterally to the ipsilateral hemitrigone), where it can be additionally tunneled in between the detrusor and mucosa (see Figure 2).

More specifically, the bladder dome is initially identified, and stay sutures or Babcock clamps are placed laterally on either side of the planned vertical midline incision. After draining the bladder, a full-thickness incision is made downward through the anterior/lateral bladder wall. A retractor is inserted through the upper pole of the incision, pulling on the bladder dome to expose the trigone clearly. An area distant from the native ureteric orifices, usually superior and lateral, is chosen, and a transverse incision is made in the bladder mucosa. A submucosal tunnel, approximately 2 cm in length, is created using a right-angle clamp or small scissors. Then the instrument is pushed outward through the muscular layer, creating an oblique transparietal opening, which can then be additionally dilated with a tunneling tool (see Figure 2A), enlarging the muscular opening to accommodate the transplant ureter. This long intramural tunnel acts as a robust anti-reflux mechanism. The ureter is then guided into the bladder (see Figure 2B), transected at an appropriate length to ensure no tension or redundancy, and its distal end is spatulated anteriorly with a 3–5 mm one-sided incision [24] (see Figure 2C). In some variations of this technique, surgeons perform two bladder mucosal incisions about 2 cm apart [40] to additionally tunnel the ureter under the mucosa (see Figure 2C,D), with the proximal incision being closed afterward using a fine absorbable suture.

Finally, the ureter is anastomosed to the bladder mucosa around the newly developed ureteric orifice, using fine absorbable sutures (see Figure 2D), with the inferior sutures also incorporating bladder muscle to anchor the ureter securely within the submucosal tunnel (see Figure 2C). At this point, a JJ stent may be placed to keep the anastomosis patent. After removing the retractor, the cystotomy incision is closed meticulously, usually using two layers of 3-0 absorbable sutures, i.e., an inferior continuous suture and a superficial discontinuous one. The bladder can subsequently be refilled intraoperatively to test for any leaks, which can be managed with additional interrupted sutures as necessary [24].

#### 3.2.2. Indications and Outcomes

The primary advantage of the L-P approach is the creation of a longer submucosal tunnel, which generally provides more effective anti-reflux protection. This can be beneficial in pediatric recipients or in patients at high risk for reflux-related complications [24,40]. Some transplant surgeons may choose an intravesical approach in cases where the recipient has known severe VUR in their native system or when bilateral kidney transplants are being performed and reflux must be minimized [30]. Additionally, the intravesical method allows direct visualization of the anastomosis from inside the bladder, which can be helpful to ensure correct mucosal apposition and to manage any bleeding from the implant site [40]. In experienced hands, the L-P technique achieves excellent outcomes as well. Older studies often considered L-P the gold standard for a reflux-proof anastomosis, and many centers have used it routinely in the past.

However, the transvesical technique is generally more time-consuming and technically complex than the extravesical method. It requires an additional distinct cystotomy, which adds another layer of closure and potential source of complications (such as bladder fistulas, hematuria and retention) [30]. Also, patients might have more catheter discomfort and bladder spasm from a larger intravesical suture line. Moreover, as the bladder must be filled and manipulated intraoperatively, in some cases, small contracted bladders may make intravesical exposure challenging. The requirement for a longer ureteric length to reach into the bladder can be a limitation if the donor ureter is short; excessive tension must be avoided. Some analyses suggest slightly higher urological complication rates with intravesical reimplantation, though results are mixed [6,40]. Given these factors, many centers have moved away from routine use of intravesical reimplantation in kidney transplants, reserving it for select scenarios (such as when an extravesical reimplant has failed and the bladder must be opened for revision).

### 3.3. Alternative Ureteroneocystostomy Techniques

**Figure 3:**
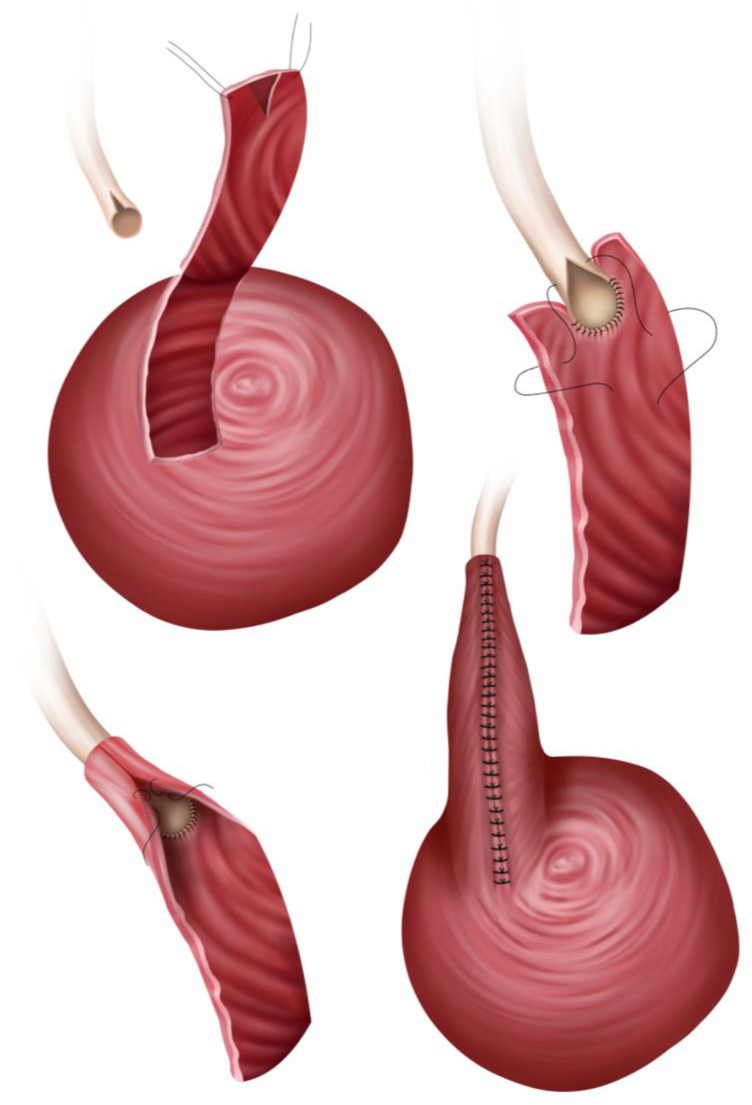
Boari flap ureteroneocystostomy technique—illustration of surgical steps. N.B.: original medical illustrations created by Marius Filip using professional graphic design software.

The psoas hitch and Boari flap techniques are surgical methods used to bridge long ureteric defects by anchoring the bladder wall and/or creating a bladder flap tube that extends cranially to meet the transplanted ureter or renal pelvis [41,42]. In the context of RT, a Boari flap UNC is an option typically reserved for situations where the donor ureter is irreparably damaged and mostly unsalvageable (e.g., with extensive ischemic necrosis), making direct UNC or ureteroureterostomy impossible due to insufficient ureteric length [1]. Essentially, the recipient bladder itself is used as a reconstructive material: a flap of bladder wall is fashioned into a tubular conduit to bridge significant ureteric graft defects (see Figure 3).

To begin, as with previous UNC techniques, prior to bladder mobilization, an antibiotic saline solution (50 mL in children, 200–300 mL in adults) is instilled through a Foley catheter to facilitate dissection. After dissecting the peritoneum from the bladder surface, if more extensive mobilization is required for an extensive defect, this can be further achieved by dividing the median umbilical ligament (urachus) and ipsilateral medial umbilical ligament, even at times extending to the contralateral medial umbilical ligament [24,41]. Herein, the overarching aim is to obtain a tension-free fixation of the bladder to the psoas muscle at a minimum of 2–3 cm cranial to the iliac vessels (psoas hitch) and a tension-free anastomosis further on (Boari flap reconstruction). If just a psoas hitch is to be performed for ureteric defects up to 5 cm, a single, superolateral, oblique, ~4–5 cm cystotomy incision will suffice. Conversely, in situations involving extensive proximal ureteric defects unsuitable for bridging with this method alone, alternatives such as the Boari flap or a modified Übelhör technique can be employed [41].

#### 3.3.1. Psoas Hitch

In psoas hitch, upon opening the bladder, the surgeon elevates the most cranial ipsilateral parietal segment with an index finger to verify that the bladder flap easily reaches the designated fixation point on the psoas muscle. The precise site of fixation is determined based on the length of the remaining proximal graft ureter, alongside the additional length required to establish a submucosal tunnel. Should tension be encountered while positioning the bladder flap, the incision is extended obliquely to increase flap length. For bladder fixation to the psoas muscle, two to three absorbable 3-0 monofilament sutures (e.g., PDS) are placed, running preferentially through the psoas tendon, and importantly, cranial to the common iliac artery and the femoral branch of the genitofemoral nerve. These sutures must fully incorporate the detrusor muscle without penetrating the bladder mucosa and remain untied at this stage [41].

The preparation of the submucosal tunnel for ureteric reimplantation requires precise surgical technique. Metzenbaum scissors or similar fine scissors are utilized to create the dissection plane between the mucosa and detrusor muscle, advancing toward the original ureteric orifice. To optimize surgical exposure, four strategic stay sutures are placed: two through the bladder wall at the tunnel entrance and two additional sutures at the planned tunnel terminus, through the bladder mucosa. Surgical efficiency is enhanced when an assistant elevates the mucosa using two forceps, facilitating expeditious and secure tunnel preparation. The optimal tunnel length should measure ~4–5 cm. It is important to note that due to tissue stretching during dissection, the final tunnel length following bladder fixation and ureter pull-through is typically shorter than initially assessed. In cases where prior inflammation, radiation therapy, or surgery causes mucosal adherence, injecting saline between the detrusor and mucosa at the intended tunnel site can ease dissection. Tunnel width is verified by spreading the scissors gently, and the distal end of the tunnel is opened transversely, excising an oval-shaped mucosal segment to prevent neo-orifice obstruction [1,41].

The ureter is then drawn into position within the tunnel by retrograde insertion of an Overholt clamp, pulling gently on the previously placed healthy ureteric stump stay suture. Subsequently, the psoas hitch sutures are securely tied. Proper alignment of the ureter should ensure a straight, tension-free course, without kinking at the bladder entry; if necessary, the tunnel entrance may be slightly widened with scissors, and any excess ureter length should be trimmed for an optimal fit at the neo-orifice. For the actual ureteric reimplantation, the ureter is spatulated at the 12 o’clock position, and two anchor sutures incorporating the ureteric wall, bladder mucosa, and detrusor muscle are placed at the 7 and 5 o’clock positions using fine absorbable glyconate monofilament sutures (5-0 or 6-0 Monocryl). The remaining ureteric orifice is closed exclusively through the ureteric wall and bladder mucosa with finer sutures (6-0 or, in infants, 7-0 glyconate monofilament). To prevent ureteric slippage, one or two absorbable monofilament sutures (5-0 to 7-0) secure the ureter’s adventitia externally to the detrusor muscle at the tunnel entrance [1,24,41].

Upon completion of the reimplantation, spontaneous urine ejaculation from the neo-orifice should be observed, indicating proper placement without obstruction; if flow is impeded, the ureter’s course is reassessed for possible kinking or obstruction. A ureteric JJ stent is inserted and may even be secured to the bladder mucosa using 4-0/5-0 glyconate or polyglytone monofilament rapid absorbable sutures. Although the classic technique usually requires the ureteric stent and a cystostomy catheter (typically a 10 Ch pigtail) to be passed through the anterior bladder wall and secured externally to the detrusor with rapidly absorbing sutures before bladder closure, to then be exteriorized at skin level, this practice does not apply generally to RT, as these additional tubes represent entry gates for pathogens in the vulnerable immunosuppressed recipients. Bladder closure is performed meticulously in two layers: the mucosa is sutured using a running 5-0 absorbable glyconate monofilament, followed by detrusor closure using a running 4-0 PDS monofilament absorbable suture. After closure, the ureteric tunnel width is checked with a small Overholt clamp. A perivesical gravity drain (12 or 16 Ch) is placed and secured externally [1,24,41].

#### 3.3.2. Boari Flap

For ureteric defects exceeding 6–8 cm that cannot be adequately addressed with the psoas hitch technique, the Boari flap provides an effective solution for achieving tension-free ureteric anastomosis [43,44]. The procedure typically employs an extraperitoneal approach similar to that described for the psoas hitch technique [41], with the main difference being the additional creation of a parietal bladder flap (versus a single incision, which lends one of its sides to be bent and used for anastomosis); this flap will then be tubularized to extend the anastomotic reach cranially, towards the ureteric stump. However, in revision surgeries, a transperitoneal approach may be more appropriate, depending on the extent of scarring and fibrosis. Conversely, in cases involving prior pelvic radiation therapy and/or multiple previous surgical interventions, identification of the ureter may become difficult and will be facilitated by locating it at its crossing with the common iliac artery or at a higher, healthier anatomical region [24,41].

As seen with all reconstructive techniques involving bladder mobilization described so far, the Boari flap procedure begins with filling the bladder via the indwelling Foley catheter. Maximum bladder mobilization is required and will be achieved, as mentioned above, through division of the median umbilical ligament (urachus) and both medial umbilical ligaments (umbilical arteries) [41]. Onward, the bladder is incised anteriorly, creating a flap typically based inferiorly, ensuring adequate blood supply from bladder vessels. Two design options exist for the flap:

1. The classical rectangular Boari flap measures ~3–4 cm in width. It is demarcated with stay sutures, with its base emerging from the posterolateral bladder wall, above the original ureteric orifice, and its distal side extending up to the contralateral anterior bladder wall [43] (see Figure 3).
2. The Übelhör modification involves making a slightly oblique incision on the affected side of the bladder, which is then extended anteriorly and distally to the contralateral side. This creates a wide rhombic flap with a broad base that can be rotated towards the psoas muscle [41].

When using a classic open approach, from this point on, the same surgical techniques seen with psoas hitch will be used for implantation. Thus, in Boari flap UNC, the same method for securing the bladder flap to the psoas muscle is used, i.e., two to three 3-0 monofilament absorbable sutures (such as Monoplus/PDS) are placed through the psoas muscle tendon. Similarly, these sutures must encompass the full thickness of the detrusor muscle without incorporating the mucosa and remain untied at this stage. Following fine parietal dissection and creation of the submucosal tunnel, an Overholt clamp is inserted retrogradely, allowing the ureter to be drawn into the tunnel via its stay suture. The psoas fixation sutures are then secured. Proper alignment is confirmed by ensuring the ureter enters the Boari flap in a straight course without kinking. The ureter is reimplanted into the bladder flap, and the bladder wall is closed using the same techniques described for the psoas hitch procedure [41].

Conversely, for endoscopic approaches (laparoscopic or robotic) [45,46], the anastomosis technique is slightly modified, as seen in Figure 3. After spatulation of the ureter, by creating an angled cut that facilitates a wider anastomotic opening, a simple submucosal space is carefully constructed at the cranial aspect of the newly created Boari flap, in a more rudimentary manner, by dissecting and incising the marginal flap mucosa [47], as seen in Figure 3. This space serves as the pathway through which the ureter will be routed into the bladder lumen. The surgeon meticulously guides the ureter through this submucosal trough, ensuring proper positioning without tension or torsion. Once the ureter has been successfully guided into position, it is secured to the mucosal layer of the flap using interrupted sutures. These sutures create precise approximation between the ureteric tissue and bladder mucosa, establishing a stable connection that promotes healing while maintaining urinary flow. The procedure continues with tubularization of the bladder flap, a critical step that transforms the flat flap into a three-dimensional conduit (see Figure 3). This reconstruction is accomplished using continuous sutures placed in two distinct layers, ensuring structural integrity and watertight closure. After flap tubularization, the surgeon addresses the remaining bladder opening [47]. This closure is similarly performed in two layers using continuous suturing techniques, methodically reconstructing the bladder anatomy while preserving function.

#### 3.3.3. Indications and Outcomes

In RT surgery, the Boari flap is seldomly used as a first-line technique [1], yet it becomes very useful in complex scenarios such as the following:

- Extensive distal ureteric necrosis: If the entire lower half of the donor ureter is necrotic, simply reimplanting it into the bladder may not be possible due to the insufficient length of viable ureter left. Thus, a Boari flap may be used, as it can reach upward to a high ureteric stump or renal pelvis, bringing up to 15 cm-long defects [44,48].
- Failed prior reimplants: In a patient who already had one or more attempts at UNC that have failed (e.g., persistent leak or stricture) and the donor ureter is now shortened, a Boari flap offers an alternative to using the native ureter [48].
- Complex ureteric injuries: Rarely, intraoperative damage to the donor ureter or recipient bladder might necessitate using such a flap in the repair, i.e., to reestablish continuity of the urinary tract in the recipient.

The Boari flap uses well-vascularized bladder tissue to replace a length of ureter, which can be advantageous when the donor ureter’s distal blood supply is compromised [1]. Because the bladder has a rich vascular plexus, the flap tends to heal well and can often succeed, even when prior ureteric repairs have failed. The technique provides a durable, one-time solution that avoids having to manipulate the native ureter or kidney, as opposed to reconstruction techniques using the native upper urinary tract (i.e., pyeloureterostomy, ureteroureterostomy, pyelopyelostomy). In the setting of early post-RT ureteric necrosis, a Boari flap could represent a useful definitive solution for salvaging the graft [49]. One case report documented a successful Boari flap repair in a renal transplant patient with distal ureteric necrosis; the flap allowed secure urinary continuity when direct reimplant was not possible, and the patient had no further complications with preserved graft function [1,49]. The use of the bladder flap inherently provides a wide-caliber conduit, lowering the risk of stricture recurrence at that site. Additionally, because the anastomosis is made to bladder tissue rather than fibrotic ureter, it may have a better chance of healing in an ischemic field.

Conversely, the Boari flap is a more extensive operation than a standard UNC. It requires sufficient bladder capacity—essentially sacrificing part of the bladder to make the flap—so it may not be feasible in patients with small or poorly compliant bladders. The bladder must be mobilized, and a large flap created, which can lead to a longer postoperative bladder healing time. Patients will need prolonged catheterization (usually 10–14 days) to allow the bladder repair to heal without parietal stress from intravesical pressure distension [1]. There is also a small risk that the flap could compromise bladder function, possibly reducing bladder volume (though in most cases bladder capacity remains adequate). Another risk is flap ischemia; if not carefully constructed, the tip of the Boari flap could become ischemic [49]. However, bladder tissue generally tolerates this well. In the transplant scenario, constructing a Boari flap can be challenging due to the presence of the transplanted graft in the iliac fossa—the surgeon must ensure that the flap can reach up to the graft ureter without tension [1].

Literature on Boari flap specifically in RT patients is limited to case reports and small series because it is an infrequent necessity [1,49,50]. The available reports suggest it can be highly effective, capable of resolving ureteric complications (such as urinary fistulas) effectively, with no recurrence and good graft function at follow-up [1]. Other series on complex ureteric reconstructions in transplants include Boari flaps as part of the armamentarium and report favorable long-term outcomes when used appropriately [49,50]. Thus, while seldom needed, the Boari flap is an important option for reconstructive transplant surgeons confronted with long ureteric defects.

### 3.4. Pyelovesicostomy

Pyelovesicostomy is an uncommon technique, rarely used in RT, where the renal pelvis of the transplanted kidney is directly anastomosed to the native urinary bladder, bypassing the ureter entirely [21]. Almost never used as a first-intention urinary reconstruction, it is usually reserved for complex ureteric lesions or cases where the graft ureter is absent or unusable along its entire length as an alternative to pyeloureterostomy. Absolute indications might include both the donor and recipient ureters being absent, necrotic or diseased so that a UNC or pyeloureteric/ureteroureterostomy cannot be performed. This rarely happens in clinical practice, yet the more common situations would be either a second RT surgery on the same side, where the first transplant’s ureter and native ureter are no longer viable options, or recurrent ureteric strictures or fistulae despite other repairs, where it is deemed better to start fresh, after wide surgical excision, within the remaining healthy tissues. Herein, albeit scarce, recent evidence has highlighted the safety and efficacy of pyelovesicostomy as an alternative treatment for complex ureteric lesions after RT, demonstrating its use in patients where standard repairs were not feasible [21,51].

#### 3.4.1. Surgical Technique

**Figure 4:**
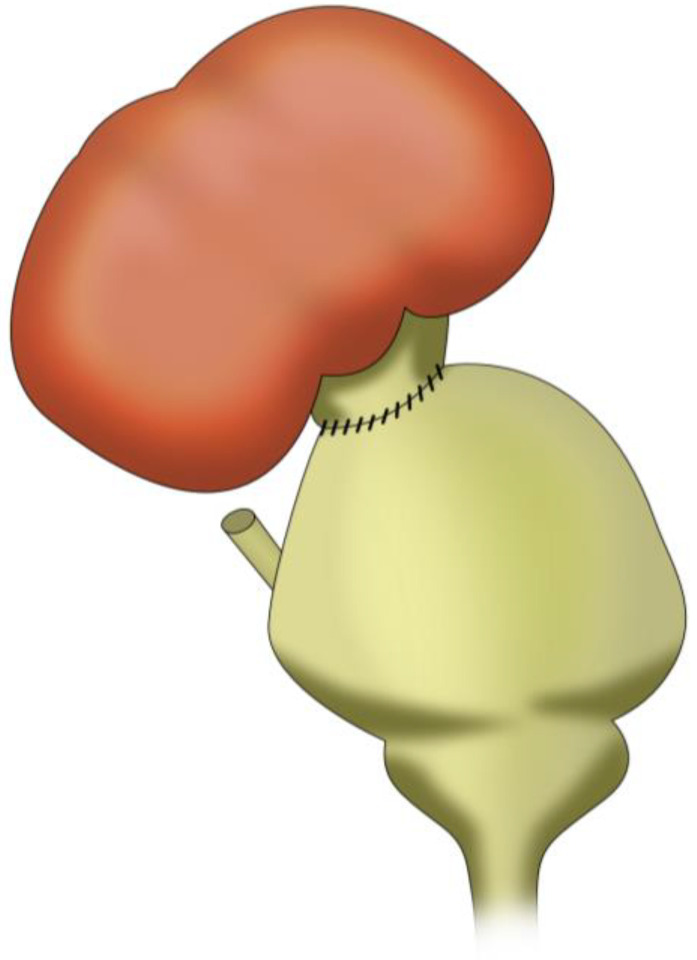
Pyelovesicostomy—illustration of final aspect. N.B.: original medical illustrations created by Marius Filip using professional graphic design software.

In pyelovesicostomy, the renal pelvis of the graft is identified and opened widely (pyelotomy) to create a generous stoma. A full-thickness cystotomy is created in the dome or anterolateral aspect of the native bladder. As seen in Figure 4, the edge of the renal pelvis is sutured directly to the bladder wall (mucosa to mucosa, in an end-to-side configuration). The anastomosis is usually augmented by bringing bladder muscle around the pelvis (similar to how a UNC would be tunneled) to provide some support and possibly reduce reflux [52]. Some surgeons might perform a bit of bladder wall infolding around the pelvis (a technique akin to a nipple valve) to mitigate reflux [52].

Pyelovesicostomy also requires that the transplanted kidney be positioned such that its pelvis can physically reach the bladder for anastomosis. Usually, the kidney is in the pelvis, so this is fine. If this is not the case, to achieve a tension-free anastomosis, the recipient bladder may be mobilized and hitched to the psoas, or a Boari flap may be constructed (as described above). Also, a JJ stent may be placed from the renal pelvis into the bladder to keep this large anastomosis patent while it heals, and a Foley catheter is left to drain the bladder and prevent distension. Essentially, the renal pelvis is treated like a short “super ureter” and attached directly, with very little tunnelization, difficult to achieve due to the wide mouth of the anastomosis.

#### 3.4.2. Indications and Outcomes

From a surgical standpoint, the procedure is conceptually straightforward, with fewer steps to the anastomosis than when using small ureteric lumens. If the bladder can be easily brought near the renal pelvis, the suture line is usually under very little tension. Another advantage is that pyelovesicostomy eliminates reliance on the ureter altogether. This can simplify the scenario when the ureter is beyond salvage. Moreover, pyelovesicostomy provides a wide opening between the graft and bladder, which will make stricture at the anastomosis site unlikely, since the renal pelvis is larger and thus less prone to scarring closed as compared to a tiny ureter.

However, for the same reason, the most notable issue with pyelovesicostomy is VUR. By creating, essentially, a direct opening of the renal pelvis into the bladder, there is no effective anti-reflux mechanism—i.e., urine can wash back into the kidney freely. Implicitly, this could predispose the graft to pyelonephritis. In practice, reflux is often well tolerated in the absence of recurrent UTIs. Surgeons have tried to create a flap or valve at the bladder anastomosis to reduce reflux [52], yet these were nowhere near as effective as the natural ureterovesical junction. Conversely, another disadvantage is that since the anastomosis is large, a leak at this level could prove to be catastrophic. Therefore, meticulous suturing and a period of bladder decompression postoperatively are essential to avoid any urine leakage in the early healing phase. Bladder dynamics also matter—a very high-pressure bladder (e.g., neurogenic bladder without augmentation) could transmit pressure to the kidney graft more readily through this open connection, potentially harming graft function over time if not addressed [51,52].

Prior literature on pyelovesicostomy in RT is sparse. Some older references in urologic surgery mention it as a possible method for salvage [53]. In a recent small series, one of the few focused on this technique, five patients underwent pyelovesicostomy with encouraging long-term outcomes—all patients had improved or stable graft function over an average of 7-year follow-up, and four out of five were living with functional grafts (one patient died 20 years postop with a still-functioning graft), with no graft lost to urological issues [51]. This suggests that once a pyelovesicostomy is healed, it can last the life of the graft with low complication rates, reaffirming that this niche technique represents a potent alternative option for surgeons facing the most challenging RT ureteric reconstructions [51].

## 4. Native Upper Urinary Tract Anastomoses

### 4.1. Pyelopyelostomy

Pyelopyelostomy refers to an anastomosis between the renal pelvis of the transplanted kidney and the renal pelvis of another kidney (typically the recipient’s ipsilateral native kidney). This is a less common reconstruction technique, essentially joining the collecting systems of two kidneys, resulting in the native ureter draining both the native and transplanted kidneys [54]. In practice, pure pyelopyelostomy (pelvis-to-pelvis) in RT is very rare, because one would need the recipient’s native kidney (on the same side) to have an accessible renal pelvis, and that native kidney is often diseased or non-functional. However, there are certain situations where a pyelopyelostomy might be considered as follows:

- In an ESRD patient with maintained diuresis who still has a functioning or semi-functioning ipsilateral native kidney and ureter and the bladder cannot be used, i.e., is difficult to identify because of pelvic scarring or shows insufficient distension for UNC, a surgeon might directly connect the transplant pelvis to the native pelvis or an upper ureteric segment, especially if the distal graft ureter vascularization seems compromised [24,29,54,55,56].
- In re-transplant scenarios where a patient has a previous transplant kidney in place, a pyelopyelostomy could theoretically connect a new graft’s pelvis to the old transplant’s collecting system (though this would be highly unusual and technically very challenging).

Because of the rarity of true pelvis-to-pelvis anastomosis, RT data on pyelopyelostomy are usually discussed alongside pyeloureterostomy. One older reference noted that pyelopyelostomy and ureteroureterostomy had been tried in some cases as alternatives to UNC [54]. If accomplished, a pyelopyelostomy would allow the transplanted kidney to drain through the recipient’s native renal collecting system and ureter, leveraging the native ureter’s natural connection to the bladder, which implicitly has the most effective anti-reflux mechanisms. Herein, it bypasses the need for a bladder anastomosis entirely [2]. In theory, if the native kidney’s pelvis is accessible and healthy, this could be a robust connection because a renal pelvis is a wide, well-vascularized structure, likely to heal well when anastomosed to another pelvis.

However, pyelopyelostomy is technically challenging and seldom feasible. The native kidney’s pelvis must be exposed, which often means a deep dissection or even partial nephrectomy if the kidney is large (like in polycystic kidney disease). If the native kidney is not functioning, its pelvis might be atrophic or scarred, making a secure anastomosis more difficult [54,55,56]. Additionally, if the native kidney is still functioning, connecting its pelvis to the transplant pelvis can create a two-kidney system with potential cross-drainage and cross-infection issues. There is a risk of reflux from one kidney to the other through the common anastomosis. In cases of discrepant function, high pressure from one kidney (or infection in one) could affect the other. Furthermore, because of these complexities, most centers will opt for pyeloureterostomy (pelvis to ureter) rather than pelvis-to-pelvis, as it is easier to anastomose the transplant pelvis to a divided native ureter (which is essentially a pyeloureterostomy) than to the native pelvis itself [24].

In fact, there is very limited direct literature on RT pyelopyelostomy. It is usually mentioned as an option in reviews of salvage techniques. One report of a complicated case described performing an “ipsilateral pyelopyelostomy” for persistent leakage after multiple surgeries, which was successful in restoring continuity, illustrating that pyelopyelostomy can be a “last resort” solution in challenging scenarios [2]. However, because it is infrequently performed, most series do not distinguish its success separately from pyeloureterostomy. In summary, while pyelopyelostomy is conceptually possible and may be life-saving in an extreme situation, pyeloureterostomy is generally preferred when using the native upper tract, as described next.

### 4.2. Pyeloureterostomy

Pyeloureterostomy is a well-established alternative for urinary reconstruction in RT. It involves connecting the renal pelvis of the transplanted graft to the recipient’s native ureter (usually the proximal or mid-ureter) as an end-to-end anastomosis. Essentially, the donor ureter is not used at all; instead, the transplanted kidney drains through the recipient’s native ureter down to the bladder. Pyeloureterostomy is typically reserved for RT cases where a standard UNC is difficult or impossible. Common indications include [9,24] the following:

1. Difficult access to the bladder: For example, if the recipient has severe pelvic adhesions, a defunctionalized bladder, or other anatomic issues (like a recent bladder augmentation or urinary diversion) that make a direct bladder implant problematic.
2. Doubtful graft ureter viability: If the transplant ureter looks poorly perfused or is damaged (e.g., limited length, signs of ischemia), the surgeon may elect to do a primary pyeloureterostomy to avoid using the compromised ureter, i.e., proactively preventing leaks/strictures.
3. Secondary (salvage) anastomosis: If a UNC was performed initially, but the patient develops a ureteric complication, a pyeloureterostomy can be employed as a rescue procedure (often preferred instead of a second UNC) [8]. Many centers consider it the preferred salvage technique for distal ureteric complications [2,8].

In a large Brazilian series, pyeloureterostomy was used in about 12.5% of kidney transplants, with 10.3% as a primary procedure in difficult cases and 2.2% as a secondary salvage for leak or stricture after initial UNC [8]. Herein, primary pyeloureterostomies had only a 3.6% leak rate and 1.7% stricture rate [8]. This underscores that while not routine, pyeloureterostomy is not exceedingly rare and is a key option in the RT surgeon’s toolkit [57].

To begin, the recipient’s native ureter (usually on the same side as the transplant, often at its iliac crossing) is identified. The anastomosis procedure begins with the completion of the posterior wall connection between the kidney transplant pelvis or ureter and either the side or the spatulated end of the recipient’s native ureter. During this process, a JJ stent is usually inserted to maintain patency, followed by completion of the anterior suture line to finalize the connection. The distal native ureter towards the bladder remains intact, so effectively the transplant drains via the native ureter into the bladder as if it were the native kidney [2,8,57].

When managing the proximal native ureter, surgeons may employ one of three distinct approaches: (1) preservation of the native kidney in its original position while utilizing the side of the native ureter for the anastomotic connection; (2) ipsilateral nephrectomy with proximal ureterectomy; (3) ligation of the proximal ureter while leaving the obstructed native kidney in place. It is imperative to note that ligation of the native ureter is contraindicated in two specific scenarios. First, in the presence of urinary tract sepsis, as this may lead to pyonephrosis of the native kidney. Second, in recipients who have previously undergone ureteric reimplantation as treatment for reflux disease, as the blood supply to the ureter may be severely compromised in these cases [24].

By maintaining the native ureter’s continuity with its kidney and creating an anastomosis between the transplant pelvis (or ureter) and the side of the native ureter, surgeons can ensure adequate blood supply to the native ureter while eliminating the risk of obstructed, hydronephrotic native kidney development [58]. Nevertheless, with the exceptions of the two contraindications mentioned above, ligation of the native ureter during pyelooureterostomy typically proceeds without complications. Conversely, when the native kidney is left in situ, even though its ureter has been rendered useless (typically tied off proximally) and will no longer drain, if it still produces urine, i.e., has residual function, it could become hydronephrotic [59]. Although a simultaneous native nephrectomy would avoid this altogether, it also adds complexity and morbidity to the surgical intervention. Thus, many simply ligate the ureter and monitor the native kidney later on (with an option to remove it if problems occur) [8].

Pyeloureterostomy offers several advantages, especially in salvage scenarios. It uses the recipient’s ureter, which generally has an excellent blood supply and is ideally implanted in the bladder, through the native uretero-vesical junction. This largely circumvents the issue of distal ureteric ischemia—since the success of the reconstruction no longer relies on the donor ureter’s perfusion [8]. As a result, the risk of a second ischemic complication is low. Studies have shown high success rates: for example, Schult et al. reported on a series of 48 pyeloureterostomies, undergone as secondary salvage surgeries for ureteric necrosis/strictures post-RT, claiming a high success rate (83% long-term graft survival with normal function), with only one graft lost to recurrent urologic complications [14]. This demonstrates the procedure’s effectiveness as a safe and simple rescue technique for distal ureteric complications, being recommended as the therapy of choice when a repeat UNC is not possible [14]. Another advantage is that the anastomosis is often easier to perform than a fresh UNC in an already fibrosed bed; sewing two luminal structures (pelvis to ureter) can be more straightforward and more accessible in such operative fields. Pyeloureterostomy also preserves the bladder (no new cystotomy or bladder suture line), which is beneficial if the bladder was already problematic or if the patient has an augmented bladder where bladder surgery is risky. Because the native ureter usually has a competent anti-reflux mechanism at the bladder, the kidney graft benefits from a near-normal drainage physiology, potentially lowering long-term UTI risk versus a direct pelvis-to-bladder draining (though comparative data on reflux incidence are limited) [2,8,51,52].

The main drawback is that the native kidney (if not removed) becomes obstructed by the ureter ligation, which can lead to complications down the line. In the Brazilian series where the native ureter was simply ligated (and the kidney left in place), about 2% of patients later required a native nephrectomy due to issues like febrile UTIs (pyonephrosis) and/or pain from hydronephrosis; risk factors for this were neurogenic bladders with augmentation or diabetes (predisposing to infection) [8]. Most of these occurred within 3–16 months after RT. In that cohort, two patients even ended up losing the transplant graft due to severe infection originating from the native kidney, and one died of uro-sepsis [8]. On the other hand, patients with only pain (often those with large polycystic native kidneys) had resolution after native nephrectomy with no further issues [8]. Thus, leaving the native kidney can pose a risk that must be monitored.

Another disadvantage is potential technical difficulty if the native ureter is not truly healthy. If the patient had urological pathology (e.g., primary reflux nephropathy), the native ureter might be scarred or unsuitable. Also, if the native ureter is short, i.e., as prior surgery partially removed it, the stump may not reach the transplant pelvis location. Usually, using the ipsilateral ureter (same side as transplant) is easiest; using the contralateral ureter would be extremely challenging.

Overall, pyeloureterostomy outcomes are generally excellent. Even as salvage after a leak, success rates were high, i.e., >90% eventually achieved fistula resolution [8], with only a small fraction (3.9%) needing further intervention for subsequent strictures [8]. In strictures treated by pyeloureterostomy, recurrences were very low (10% needed ongoing stenting) [8]. These data confirm that pyeloureterostomy is a reliable solution. It has become a preferred method for secondary repair in many centers because it offers a one-and-done fix with good long-term patency [8,59,60,61].

### 4.3. Ureteroureterostomy

Ureteroureterostomy in the RT context typically refers to an anastomosis between the donor ureter and the recipient’s native ureter. It differs from pyeloureterostomy mainly in that the donor ureter (not the pelvis) is anastomosed, usually end-to-side or end-to-end. Functionally, it achieves the same goal: routing the transplant urine into the native ureteric conduit. This is why many authors use “ureteroureterostomy” as a broad term that could include pyeloureterostomy. Moreover, its indications also overlap heavily with those of pyeloureterostomy [24]. If the donor ureter is intact enough, but one prefers to use the native ureter’s connection to the bladder (for reasons discussed above), a ureteroureterostomy can be performed. This might be considered when

- The donor ureter is still viable in part, but the very distal end or implantation site is problematic (so one can join the donor ureter to the native ureter a little higher up instead of to the bladder).
- The native ureter is easily accessible and healthy, making a ureter-to-ureter anastomosis straightforward.
- As a salvage for a distal stricture, some have managed short strictures by excising the damaged segment and performing an end-to-end anastomosis between the remaining donor ureter and the native ureter. However, more commonly surgeons go up to the pelvis of the transplant because it is a larger target; thus, ureteroureterostomy per se is slightly less common than pyeloureterostomy [2,8,24].

**Figure 5:**
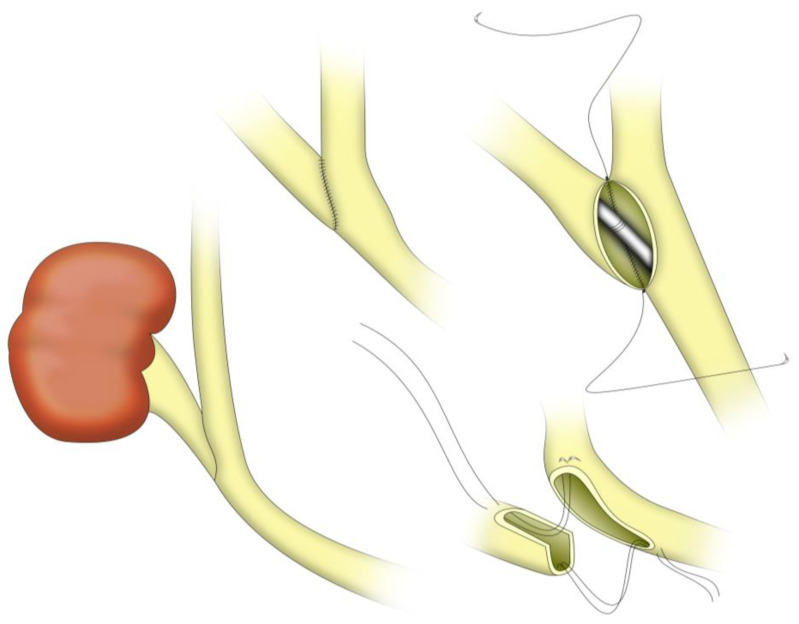
Ureteroureterostomy technique—illustration of surgical steps. N.B.: original medical illustrations created by Marius Filip using professional graphic design software.

The native ureter is usually transected at a convenient level, often above its lower third. The donor ureter is trimmed to healthy tissue. An end-to-end anastomosis is performed between the two ureters over a JJ stent [48]. Alternatively, as seen in Figure 5, an end-to-side technique can be used, where the native ureter is slit open on one side and the donor ureter is implanted into that opening, creating a calibrated wide anastomosis [24,62]. The latter might be useful if the native ureter is larger or one wants to avoid complete transection (to preserve native kidney drainage, as needed). Conversely, in the end-to-end scenario, the native ureter proximal to the anastomosis may be simply ligated, or native nephrectomy may be performed, similarly to the pyeloureterostomy protocol [48].

As seen in Figure 5, the end-to-side anastomosis technique commences with the strategic placement of 5-0 monofilament stay sutures, positioned side-by-side in the recipient ureter, delineating the predetermined site for longitudinal ureterotomy. A precise incision is then made in the native ureter using a fine scalpel (no. 15 blade) and further extended using Potts scissors to create an appropriately sized match to the donor ureter opening and reveal the JJ stent mounted preoperatively. To establish initial alignment, similar absorbable 5-0 sutures are positioned at both extremities of the recipient ureterotomy and secured to the corresponding heel and toe positions of the donor ureter (see Figure 5).

The posterior wall anastomosis proceeds with the donor ureter being sewn to the medial wall of the recipient ureter from within the lumen. During this phase, an unscrubbed assistant withdraws the recipient ureteric catheter until its open end becomes visible in the partially completed anastomosis. A guidewire is then passed through the end of the recipient JJ catheter, and then this stent is withdrawn completely. Herein, the graft stenting process involves passing a new JJ stent over the guidewire and into the bladder. A precautionary suture may be left attached to the distal curl to facilitate repositioning should the curl retract up the ureter. Following removal of the wire, confirmation of the distal curl’s position is achieved when the anesthesiologist fills the bladder through the Y-connector hooked up to the Foley catheter by clamping the outflow and opening the inflow. To complete the stent placement, a guidewire is passed through a JJ stent side hole to straighten the proximal curl, allowing it to be advanced up the donor ureter and into the renal pelvis. The guidewire is subsequently removed after proper positioning is achieved. The ureteroureterostomy is finalized by completing the running suture line using 5-0 monofilament absorbable suture material, creating a secure and watertight connection between the donor and recipient ureters [62].

Ureteroureterostomy allows use of the native anti-reflux mechanism and blood supply, similar to pyeloureterostomy. If the donor ureter has enough length and good blood flow except at the very end, this technique saves the surgeon from having to perform more complex alternative UNCs (Boari flap with psoas hitch), while also reducing the extent of dissection (necessary for pyelopyelostomy and/or pyeloureterostomy) and preserving a length of donor ureter for future revisions, yet still providing a well-vascularized anastomotic partner (the native ureter) [48,63]. It can be a very clean solution in these cases, where donor ureteric length is detected intraoperatively as insufficient to comfortably reach the bladder, as the surgeon can directly approach the native ureter, which might be closer [24,48]. Another scenario is pediatric RT: in small children with weak bladder outlets, surgeons sometimes anastomose the graft ureter to a native ureter, thus avoiding the bladder entirely. This can also potentially preserve the option of future ureteric reimplantation, seeing as the native ureter insertion is untouched.

Conversely, the site of ureter-to-ureter anastomosis could be a potential point of stricture if not performed properly, as the two ureters might not have identical diameters. Herein, the blood supply of the donor ureter still matters; if it is questionable, pyeloureterostomy might be preferable, as the pelvis will have better blood flow. Also, if the donor ureter is long and mostly healthy, ureteroureterostomy may be appropriate, but if only a short stump is healthy and usable, anastomosing it to the native ureter might prove to be quite tenuous. Essentially, ureteroureterostomy shares the same pros/cons as pyeloureterostomy, with the nuance that it keeps the anastomosis at the donor ureter level [8,24,48,63].

In the literature, results of ureteroureterostomy are typically not separated from pyeloureterostomy [24]. When performed properly, one would expect similar success—a well-vascularized native ureter should heal well to a healthy donor ureter. In terms of evidence, earlier RT eras saw attempts at ureteroureterostomy, with small case series or case reports noting favorable outcomes [64,65,66]. For instance, Whelchel et al. (1975) reported, in a single-center study, that pyeloureterostomies and/or ureteroureterostomies were the primary urinary tract reconstructive procedures used in 114 of 132 consecutive RTs performed between September 1967 and December 1973 [67]. In this cohort, urinary leaks were the primary complication (~8%) [67], indicating that as long as the surgical technique is sound, outcomes are acceptable. Moreover, it was noted that after 1970 they had only 4 anastomotic leaks in 79 consecutive pyelo/ureteroureterostomies [67], showing improvement as techniques refined. In modern times, if a surgeon chooses ureteroureterostomy, it is often at initial RT in specific patients (like pediatric or complex anatomy), and the limited evidence available shows that patient and graft survival are not compromised by using this method [68,69].

While most transplant centers continue to use ureteroureterostomy primarily as a salvage technique for cases with ureteric complications, limited but compelling literature suggests this approach may yield lower reoperation rates than standard techniques. This advantage may stem from the fact that distal ureter vascularization issues in the donor organ—which often lead to stenosis or necrosis and subsequent urine leakage—are bypassed entirely when utilizing the recipient’s healthy native ureter [70]. Additionally, despite the anti-reflux mechanisms built into traditional UNC techniques, VUR remains a significant clinical concern with those approaches. Such reflux increases the risk of recurrent UTIs and pyelonephritis, potentially compromising long-term graft function [57].

A common hesitation among surgeons regarding this technique centers on the perceived limitation of remedial options should complications arise, as the native ureter would no longer be available for subsequent repair. However, clinical experience demonstrates the opposite effect. The preservation of native ureter continuity actually facilitates endoscopic catheterization and provides straightforward access for standard endourological procedures such as ureteroscopy when investigating or treating urological events post-RT [68].

Overall, the end-to-side ureteroureterostomy technique offers several clinical advantages: earlier removal of bladder catheters (typically on the first postoperative day), reduced UTI risk, elimination of routine JJ catheter placement, and the ability to perform endoureteric manipulations through natural anatomical pathways—similar to procedures in non-RT patients. This approach overcomes the access challenges sometimes encountered with bladder dome implantation techniques [68].

Furthermore, this streamlined technique reduces operative time, offers greater reproducibility across surgeons with varying experience levels, and preserves the recipient’s normal anatomical structures—all compelling reasons to reconsider end-to-side ureteroureterostomy as a primary rather than salvage technique in RT.

## 5. The Use of Bowel in Urinary Reconstruction

Ureteroenterostomy refers to an anastomosis of the ureter to an intestinal segment. In RT, this usually means implanting the donor ureter into a pre-existing urinary diversion (like an ileal conduit or orthotopic neobladder) or into an augmented bladder’s bowel segment. It can also mean creating a new bowel conduit for the transplanted graft if the bladder or native upper urinary tract are not usable. Essentially, ureteroenterostomy is when urine from the renal graft is diverted to or through the bowel.

The primary indication is a recipient who does not have a normal functioning bladder for the transplant to drain into. Examples include post-cystectomy patients with severe neurogenic bladders; with very small bladder capacity; or with other complex bladder diseases that cannot be corrected before or during RT. In some cases, surgeons will actually perform a simultaneous or prior urinary diversion to protect the transplant.

From a surgical perspective, anastomosis to a bowel can be slightly tricky: bowel tissue is more fragile than bladder and needs delicate suturing to avoid tearing. If multiple prior surgeries exist, finding and mobilizing the conduit or augmented bladder may be time-consuming. Notwithstanding some sporadic technical success shown with laparoscopic or robotic approaches in these complex cases, typically open surgery is performed due to scar tissue from previous interventions.

If an ileal conduit exists, the donor ureter can be implanted into the conduit using a standard Bricker technique (the end of the graft ureter is spatulated and implanted into the side of the ileal loop, usually over a stent, with a maturing sero-muscular stitch [71]). If multiple ureters (like bilateral transplants) or a small conduit that already has native ureters, careful planning is needed to avoid tension on the blood supply of the conduit. In the case of an augmented bladder (enterocystoplasty), there are two approaches:

1. The classical approach: implant the transplant ureter into the native bladder portion (to take advantage of some anti-reflux capacity, both inherent and surgically constructed). Many sources suggest performing it this way [72].
2. The alternative approach: implant directly into the bowel segment of the augmented bladder [52].

The transplanted ureter can be implanted into either the native bladder neck region or the bowel patch. Traditionally, surgeons aimed for the native bladder trigone if possible. But if the augmentation is extensive and the bladder neck is small or scarred, implanting into the bowel patch may be the only way. As reported by Tan et al. (2019), direct bowel segment implantation with an anti-refluxing suture technique is feasible and produced no implantation failures in their series [52]. In their technique, they sutured the ureter to the mucosa of the bowel patch and then plicated the bowel around it to prevent reflux [52]. They reported performing this in 7 patients with augmented bladders, all of whom had good outcomes (no ureteric complications, low infection rates). They concluded that implanting into the bowel segment can be safe and effective [52]. However, some precautions should be considered, as it is customary to stent the ureter longer in augmented bladders (some leave stents ~3–6 weeks) and keep a Foley catheter for an extended period (7–14 days) to ensure low pressure while the anastomosis heals. The presence of bowel mucosa means mucus can clog catheters; flushing the catheter regularly is important.

For a new conduit creation, the same standard Bricker technique is generally preferred [71,73]. The distal end of an isolated segment of ileum, usually 15–20 cm long, harvested in its isoperistaltic orientation, at least 15–20 cm away from the ileocecal junction, is exteriorized transabdominally and fashioned into a cutaneous stoma, while the proximal end is used for the ileoureteric anastomosis. The ureteric stump of the graft, possibly alongside the native ureters if diuresis is maintained, will be spatulated and anastomosed along the antimesenteric side of the conduit, using 4-0 polyglactin sutures and one of the multiple techniques described for ileoureteric anastomosis: either directly altogether (the Wallace technique and its variations) or separately (at least 1–3 cm apart from each other—the Nesbit and Bricker techniques) [73]. Moreover, as the transplanted ureter is usually short, sometimes even the renal pelvis may be used to gain access into the conduit.

Ureteroenterostomy allows patients with abnormal lower urinary tracts to still receive transplants with secure urinary drainage. Research investigating uretero-intestinal anastomosis in RT reveals important clinical implications. Historically, some concern existed that connecting a transplanted kidney to the bowel could lead to higher infection and electrolyte issues. In a seminal 1990 investigation, Nguyen and colleagues demonstrated inferior graft survival rates and elevated complication frequencies when donor ureters were implanted into intestinal segments of enterocystoplasty compared to native bladder tissue [74]. The documented complications encompassed a spectrum of adverse events, including anastomotic leakage, stenotic development at the anastomotic site, calculus formation, urosepsis, and wound-related complications such as infection and dehiscence [74]. Herein, it is important to note that attributing complications specifically to the ureteroenteric implantation presents significant methodological challenges. This difficulty arises because the procedure constitutes one component of RT—a complex surgical intervention associated with substantial morbidity and mortality. Surgical outcomes are influenced by multiple variables beyond technical execution, particularly patient-specific factors including age and pre-existing health status.

Despite comprehensive preoperative optimization strategies, RT patients frequently represent suboptimal surgical candidates due to their chronic disease state. The prolonged uremic condition can adversely affect nutritional status, which subsequently impacts postoperative recovery trajectories. Additionally, many candidates have undergone previous abdominal procedures, potentially introducing technical complexity to the transplantation process [52].

Furthermore, gastrointestinal tissue presents inherent limitations when utilized for bladder augmentation. Its application in functionally or structurally compromised bladders is associated with several consequential complications. These include metabolic derangements, particularly acidosis, urinary stone formation, and—of greatest clinical concern—malignant transformation. The pathogenesis of malignancy in this context involves multiple factors, with urinary stasis, chronic bacteriuria, and resultant persistent inflammation representing the principal contributory mechanisms [52].

Conversely, more recent studies have consistently shown that, reassuringly, augmented bladder recipients may in fact associate graft and overall survival rates comparable to regular RT patients [52,75,76,77]. Reassuringly, not even a decade later from previous early detrimental reports, Yamazaki et al. noted that aside from UTIs, no other complications were observed in their small series, and all patients achieved a large, low-pressure bladder post-RT, continent with clean intermittent catheterization [76]. They concluded that transplantation into extensively reconstructed bladders can be performed safely with good success rates, though UTI remains a major consideration [76].

More recently, in one of the largest investigations of its kind, Surange et al. (2003) reported on a series of 59 RT cases with ureteric implantation into ileal conduits, operated over a 22-year period, which showed a graft survival of 63% at 5 years and 52% at 10 years, identical to patients transplanted with normal bladder drainage [75]. Patient survival was also similar [75]. Furthermore, pediatric data similarly show no difference in graft survival, though UTI rates can be higher [77]. In corroboration, this evidence seemingly demonstrates that using a conduit does not inherently shorten graft life in RT. Therefore, an augmented bladder per se should not be viewed as a contraindication to RT but rather as a situation requiring specialized management.

In fact, performing reconstruction or diversion prior to RT may aim to optimize the urinary tract, i.e., ensure an ideal low-pressure system for the graft [76]. Thus, ureteroenterostomy actually expands RT access to patients who otherwise might not be candidates due to urinary tract issues. Even so, patients with bladder augmentation usually have it due to a prior small or high-pressure bladder (such as neurogenic bladder from spina bifida). Prior to RT, it is critical that the augmented bladder be compliant and of adequate capacity to avoid high-pressure urine refluxing into the graft. Urodynamic studies are generally required as part of the preoperative assessment. If the augmented bladder is still unsafe, further reconstruction or a switch to an external diversion might be warranted.

On the other hand, we must note that even though graft survival is generally equivalent in RT patients with urinary diversions, they must endure a higher complication burden, mainly urological and infectious. In the ileal conduit series above (Surange et al.), it was also reported that 21% of patients had surgical complications directly attributable to the conduit, and an additional 39% had complications where the conduit was a contributing factor [75]. These included things like stoma problems, uretero-ileal strictures (one graft lost from a ureteroenteric stricture), and stones. Symptomatic UTIs occurred in 65% of patients with an ileal conduit, a much higher fraction than in typical RT patients [75]. However, importantly, those UTIs did not usually cause graft loss [75]. Patients can have bacteriuria chronically due to the bowel and need to be educated on stoma care or catheterization technique. Metabolic issues can arise also: absorbing urinary solute through an ileal segment can cause metabolic acidosis or electrolyte imbalances (like hyperchloremic acidosis common in ileal diversions), which might need medical management. Implicitly, these patients should be monitored for metabolic acidosis and for any signs of urinary sepsis. Frequent monitoring of urine cultures is also warranted.

If the transplant ureter is implanted into an augmented bladder, the same infection risk applies—colonized mucus-producing epithelium can seed bacteria. Indeed, post-RT pyelonephritis was noted frequently in augmented bladder RT patients (3 in 4 cases), although graft function remained satisfactory [76]. Aggressive surveillance and treatment of UTIs is required in these patients. Another issue is malignancy risk: long-standing chronic inflammation in a diversion or augment can lead to rare bowel or urothelial cancers. Transplant immunosuppression might potentiate that risk slightly. Obviously, careful long-term monitoring is advised, with hematuria being a significant cause for concern.

Long-term management of recipients with complex urinary reconstructions requires systematic protocols addressing both infectious complications and functional maintenance of reconstructed systems [52,75]. Antibiotic prophylaxis strategies play a crucial role in preventing recurrent UTIs that can jeopardize both graft function and patient safety. Continuous low-dose prophylaxis using agents such as trimethoprim–sulfamethoxazole or nitrofurantoin is indicated for recipients with neurogenic bladders, bowel-based reconstructions, or recurrent symptomatic infections despite optimal hygiene and catheterization techniques [76,77]. Regular surveillance with urine cultures every three to six months enables early detection of bacterial resistance patterns and guides appropriate modification of prophylactic regimens [75].

Self-catheterization represents a fundamental component of long-term care for recipients with augmented bladders, continent diversions, or catheterizable channels [52,76]. Successful programs require comprehensive initial training in aseptic technique, recognition of complications, and emergency management protocols for catheter blockage or inability to catheterize [13]. The unique challenges of managing immunosuppressed recipients with complex urinary reconstructions necessitate heightened vigilance for infectious complications and malignant transformation, with regular monitoring including surveillance for UTIs, assessment of renal function, and screening for malignancy in recipients with long-standing bowel-based diversions [74,77].

Overall, it seems that despite the early complication burden, with careful management, these patients seemingly fare as well as regular RT patients long-term [75,76]. Infection prophylaxis, through extended peri/postoperative use of suppressive antibiotics and aseptic technique for all invasive maneuvers (especially regarding clean intermittent catheterization in the long term), is crucial. Equally important are adequate surgical technique and vigilant postoperative care. With these prerequisites, ureteroenterostomy can represent a safe alternative in selected patients, thus expanding the addressability of RT.

## 6. Conclusions

Ureteric complications represent an enduring challenge in RT despite significant surgical advances. While incidence has decreased with improved techniques and routine stenting, these complications continue to affect ~2–5% of recipients and significantly impact graft function if not promptly addressed.

The pathogenesis is multifactorial, with ischemic injury to the distal ureter being predominant. The graft ureter’s unique vascular vulnerability—receiving blood supply solely from inferior renal artery branches—makes it susceptible to compromise during procurement, bench preparation, and implantation. This underscores the critical importance of meticulous surgical technique, preserving periureteric tissues, and adequate blood supply.

Ureteric leaks and strictures rooted in donor ureter ischemia demand prompt surgical response. Transplant surgeons possess a robust armamentarium of reconstruction techniques. Standard extravesical UNC (L-G) provides reliable first-line management, preferred for simplicity, reduced operative time, and equivalent or lower complication rates versus transvesical L-P, which remains an effective anti-reflux alternative. Complex cases require mastery of alternative techniques.

Pyeloureterostomy and ureteroureterostomy utilizing the native ureter have demonstrated excellent outcomes as salvage procedures, with high success rates and durability. These techniques effectively bypass compromised donor ureter segments and leverage the native ureter’s robust blood supply and established anti-reflux mechanism. For extensive ureteric defects, the Boari flap with psoas hitch provides a reliable solution using well-vascularized bladder tissue. Although rarely needed, pyelovesicostomy and ureteroenterostomy remain important options in the reconstructive arsenal for specific challenging scenarios.

Comparative analysis reveals no single method fits all scenarios—optimal approaches are case-dependent. Surgeons must weigh donor ureter viability, recipient bladder/ureter condition, and complication timing. Early post-transplant leaks from distal necrosis may require abandoning the distal ureter for pyeloureterostomy, while late strictures in healthy bladder systems might benefit from tailored reimplantation or endoscopic dilation. Patients with reconstructed/diverted urinary tracts require careful planning, yet proper technique achieves comparable graft outcomes to normal bladders.

Contemporary management of ureteric complications involves a multidisciplinary approach combining surgical, endourological, and radiological interventions. Early diagnosis through vigilant monitoring and prompt intervention is essential to prevent irreversible graft damage. While endoscopic management may serve as a temporary measure, definitive surgical reconstruction is often necessary for lasting resolution of significant ureteric leaks or strictures.

The complexity of ureteric reconstruction in RT demands coordination between transplant surgery, urology, interventional radiology, and nephrology, with each discipline contributing essential skills to comprehensive patient care [3,23,24]. Transplant surgeons provide the surgical foundation, contributing their expertise in immunosuppression and graft physiology, while urologists become crucial when faced with complex bladder dysfunction, advanced reconstructive techniques, and long-term management of augmented or diverted urinary systems [52,76]. Interventional radiology plays an increasingly important role in both diagnostic evaluation and therapeutic intervention, providing temporizing drainage procedures, percutaneous access for complex reconstructions, and minimally invasive management options that can bridge patients to definitive surgical repair [11,17]. Nephrology input ensures optimal immunosuppressive management during the perioperative period, balancing infection risk with rejection prevention, while also managing the metabolic consequences of urinary diversions [5]. Specialized nursing expertise in complex catheter management, stoma care, and patient education protocols proves essential for long-term success, particularly in recipients with augmented bladders or urinary conduits [13,52].

The volume–outcome relationship for complex ureteric reconstructions suggests that centers performing <50 transplants annually should consider early consultation or referral of challenging cases to high-volume centers with established expertise in these techniques [9,25]. This approach ensures that recipients receive optimal care while maintaining the safety and efficacy that characterize successful ureteric reconstruction programs [6,23]. The integration of specialized expertise, systematic protocols, and evidence-based decision-making creates the foundation for successful management of even the most complex ureteric complications in RT [24].

Future directions in the field should focus on further surgical technique refinement, novel ureteric vascular preservation approaches, and standardized complication. Prospective comparative studies evaluating outcomes of different reconstruction techniques could provide stronger evidence to guide clinical decision-making.

**Figure 6:**
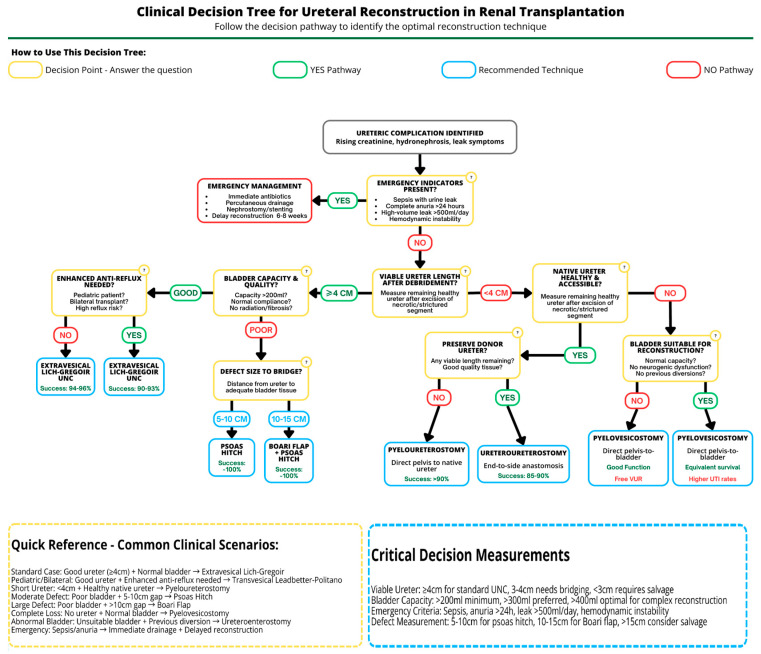
Clinical decision tree algorithm for systematic selection of ureteric reconstruction techniques in renal transplantation. Clinicians follow sequential binary decision points based on emergency indicators, viable ureter length, bladder quality, and native ureter status to reach technique-specific recommendations. Success rates represent contemporary outcomes from high-volume transplant centers.

To facilitate clinical decision-making, we have developed a comprehensive algorithm (see Figure 6) that systematically integrates complication-specific factors, recipient characteristics, and technique-specific requirements to guide optimal reconstruction selection. This evidence-based framework addresses the critical need for standardized approaches to managing ureteric complications in RT, providing clear decision thresholds and contraindication criteria for each reconstructive technique.

Ureteric complications, while formidable, can be effectively addressed with the spectrum of urinary reconstruction techniques reviewed in this paper. Key tenets include ensuring a well-vascularized anastomosis, maintaining urinary drainage, and selecting techniques aligned with patient needs and surgical context. Contemporary literature demonstrates encouraging outcomes—most patients, even those requiring complex reconstructions, go on to have excellent graft function and long-term survival. By maintaining a high index of suspicion for complications and being prepared with flexible surgical strategies, clinicians can minimize the morbidity of ureteric issues in RT. This tailored, patient-centered approach—as evidenced by the various techniques and results discussed—underscores the modern surgical ethos in RT: successful graft outcomes depend not only on immunological management but also on meticulous surgical planning and execution. A comprehensive understanding of the various urinary reconstruction techniques equips the transplant surgeon to address these challenges successfully, ultimately improving graft longevity and patient outcomes.

## Data Availability

The data presented in this study are available on request from the corresponding author.
